# Fight for carbon neutrality with state-of-the-art negative carbon emission technologies

**DOI:** 10.1016/j.eehl.2022.11.005

**Published:** 2022-12-05

**Authors:** Jiaju Fu, Pan Li, Yuan Lin, Huitong Du, Hongzhi Liu, Wenlei Zhu, Hongqiang Ren

**Affiliations:** a State Key Laboratory of Pollution Control and Resource Reuse, State Key Laboratory of Analytical Chemistry for Life Science, The Frontiers Science Center for Critical Earth Material Cycling, School of the Environment, School of Chemistry and Chemical Engineering, Nanjing University, Nanjing 210023, China; b Chinese Society for Environmental Sciences, Beijing 100082, China

**Keywords:** Carbon neutrality, Renewable energy, Negative carbon emission, Carbon utilization, Carbon footprint reduction, Climate change mitigation

## Abstract

After the Industrial Revolution, the ever-increasing atmospheric CO_2_ concentration has resulted in significant problems for human beings. Nearly all countries in the world are actively taking measures to fight for carbon neutrality. In recent years, negative carbon emission technologies have attracted much attention due to their ability to reduce or recycle excess CO_2_ in the atmosphere. This review summarizes the state-of-the-art negative carbon emission technologies, from the artificial enhancement of natural carbon sink technology to the physical, chemical, or biological methods for carbon capture, as well as CO_2_ utilization and conversion. Finally, we expound on the challenges and outlook for improving negative carbon emission technology to accelerate the pace of achieving carbon neutrality.

## Highlights

• State-of-the-art negative carbon emission technologies are reviewed and summarized.
• The challenges and prospects of each technology are given.
• Since both capture and conversion are relatively energy-consuming, the intelligent design of combined systems can significantly reduce costs and operational steps, which may be the focus of future negative carbon technology development.

## Graphical abstract

**Figure:**
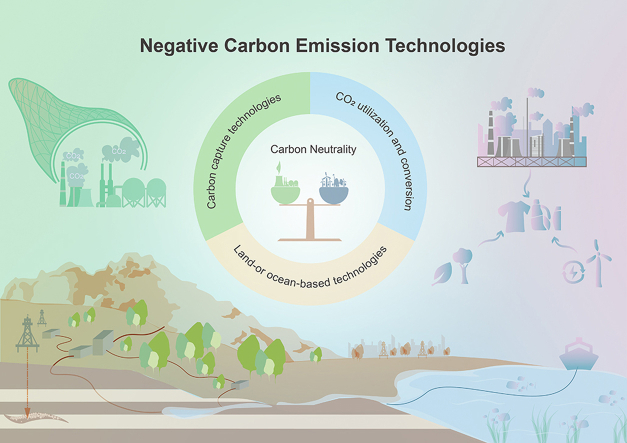

## 1 Introduction

The ever-increasing worldwide fossil fuel consumption has caused a rapid increase in atmospheric concentrations of CO_2_ from a pre-industrial value of 280 ppm to 415 ppm by 2021, resulting in a severe greenhouse effect [[1], [2], [3]]. Global temperatures have risen by an average of 1.07 °C and, over the past four decades, have been the warmest for any period since 1850 [3]. However, in 2021, 330 billion tonnes of CO_2_ emissions from anthropogenic activities remained at high levels, of which more than three-quarters came from burning fossil fuels [4]. If left unchecked, the global warming caused by the greenhouse effect would lead to more severe environmental crises, including catastrophic melting glaciers, rising sea levels, frequent extreme weather, and biodiversity loss [3,5,6].

Global warming could be attributed to the increased absorbance of solar radiation by greenhouse gases (CO_2_, methane, etc.) [7]. Reducing excessive greenhouse effects could decrease solar energy absorbance, thus mitigating climate change. Several promising approaches have been promoted recently to reduce greenhouse gas emissions. Developing low-carbon and zero-carbon technologies, including renewable energy technologies such as solar, hydropower, bioenergy, wind, geothermal, and tidal power, along with energy storage and energy-efficient technologies, could solve global warming from the source [[8], [9], [10]]. However, fossil fuels still cover about 80% of current worldwide energy needs, which might play an essential role over a decade or so [9]. There is still a long way to go toward developing highly efficient energy systems with zero-carbon emissions. On the other hand, other perspectives proposed that global warming may also result from the imbalance between the radiation imported into the Earth and that reflected into outer space. Thus a solar radiation management strategy was developed to combat the Earth’s warming by reducing the percent of sunlight received by the Earth, such as space mirrors, sulfate aerosols, and cloud whitening [[11], [12], [13], [14]]. This approach may succeed in coping with climate emergencies. For example, the melting of the Greenland ice sheet constantly releases large amounts of methane. Adopting this strategy could obtain a good result in a short time as compared with other methods [13]. Although the solar radiation management pathway could temporarily stabilize global temperatures by reducing the contribution of other factors to increased temperatures to offset the contribution of increased CO_2_ concentrations, the issues of the increasing greenhouse gas concentrations accumulated in the atmosphere still exist [14]. In addition, the impact of solar radiation reduction on other non-climatic factors (such as plant photosynthesis, ecosystem stability, and even human health) is unknown [11,14].

To date, countries worldwide have made many efforts to deal with global warming. At the recent 26th United Nations Climate Change Conference of the Parties (COP26), nearly 200 parties finally reached the Glasgow Climate Pact, which further clarified the reduced greenhouse gas emissions to keep the average temperature rise within 1.5 °C and avoid the catastrophic consequences of exacerbated climate change [5,15]. Holding the global temperature rise within 1.5 °C above the pre-industrial levels (currently 1.2 °C) is the most ambitious goal of the Paris Agreement [15,16]. With that aim, a 55% cut is required in global CO_2_ emissions by 2030 from 2010 levels, and net emissions of greenhouse gases become net-zero by 2050, which means the balance between anthropogenic emissions and removal of greenhouse gas in the second half of the 21st century [3,15]. For this reason, countries worldwide need to formulate policy planning and objectives on climate and energy based on the state of respective national energy systems and take positive measures to fulfill their commitments to reduce emissions and mitigate global climate change. In addition to vigorously developing renewable energy and energy-efficient technologies, the intergovernmental panel on climate change (IPCC) assessment reports highlight the crucial role of negative carbon emission technologies in achieving the aims of the Paris Agreement [5,[17], [18], [19]]. Negative carbon emission technologies could remove CO_2_ from the atmosphere and store or use it to offset carbon emissions that are difficult to reduce.

**Fig. 1:**
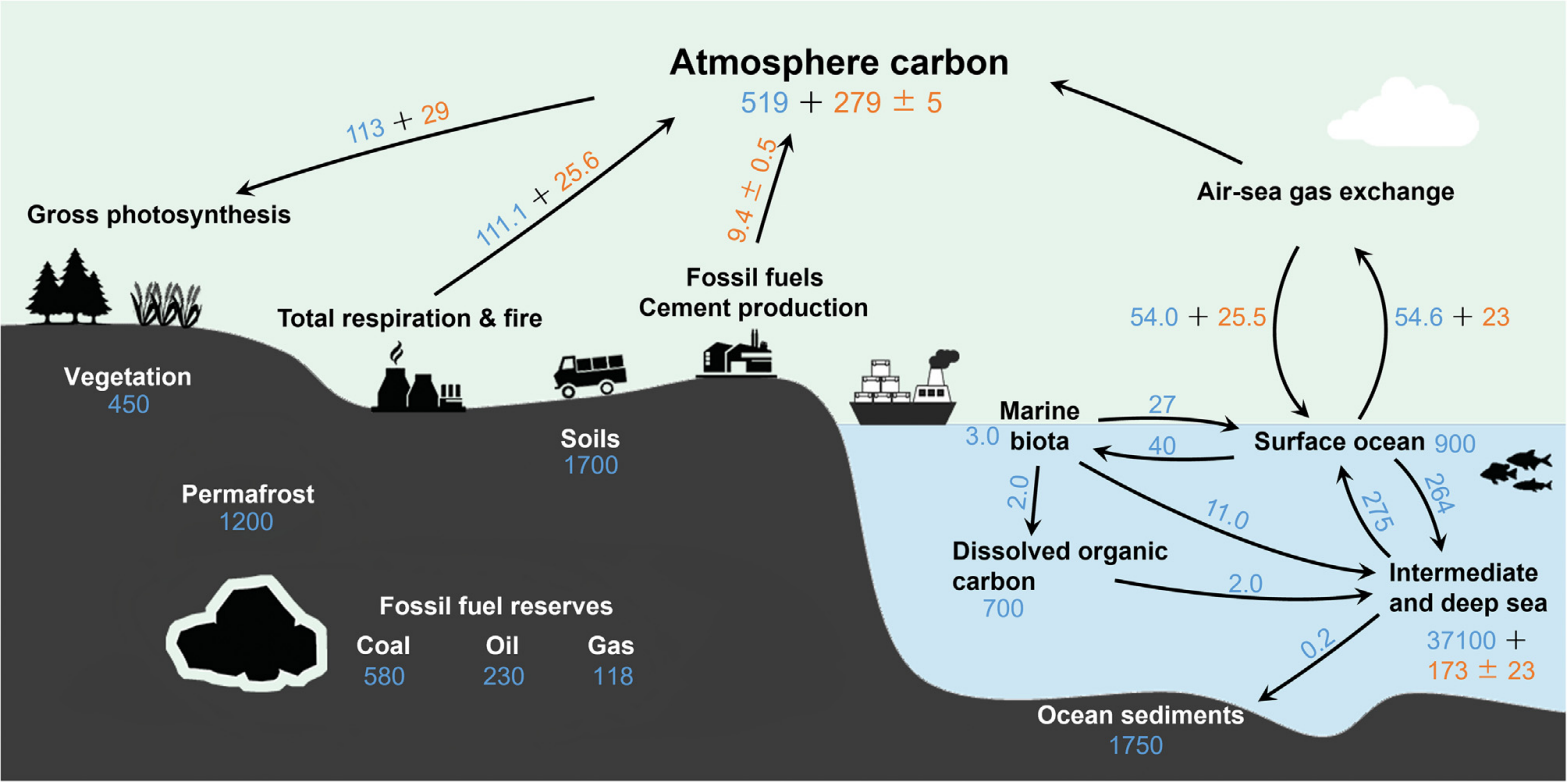
A schematic diagram of the main Earth’s carbon cycle shows the movement of carbon between the atmosphere, lithosphere, hydrosphere, and biosphere. The blue numbers represent natural fluxes or stocks, and the orange numbers represent anthropogenic fluxes or stocks (Flux: Billion tonnes of Carbon per year, PgC/yr; Stocks: Billion tonnes of Carbon, PgC). Data source: IPCC Sixth Assessment Report, Work Group 1, Chapter 5 (2021).

In addition to the combustion of fossil fuels, CO_2_ comes from the respiration of animals and plants and the decomposition of animal carcasses by microorganisms as well. The vast majority of carbon in nature is stored in crustal rocks, and the rest is in the atmosphere, soil, ocean, plants, and fossil fuels. The carbon exchange and circulation processes in the biosphere, lithosphere, hydrosphere, and atmosphere are called the carbon cycle, as shown in Fig. 1. In the natural carbon cycle, green plants and marine photosynthetic organisms consume CO_2_ to synthesize organic matter for removing CO_2_ from the atmosphere. The essence of negative carbon emission technologies is to enhance the sink of atmospheric CO_2_ and weaken its source.

In this review, we begin with a summary of recent advances in land- or ocean-based technologies and methods, whose principle is largely based on photosynthesis. Unfortunately, these natural process-based carbon sinks still have limited CO_2_ capture capacity due to limited land and ocean resources, even with artificially enhanced interventions, taking a long time to have a significant effect. Given the current urgency of climate change, the above technologies need to be combined with other negative carbon technologies based on physicochemical or biological mechanisms, mainly including carbon capture and storage technologies and utilization and transformation technologies. The most recent results of each technique are now reviewed and summarized. Then the challenges encountered and perspectives for future development are outlooked at last.

## 2 Land- or ocean-based technologies

Atmospheric CO_2_ can be maintained at a certain level based on natural processes such as photosynthesis, soil and ocean uptake, etc. With the increase in human activities, the natural carbon sink is not enough to maintain the balance of CO_2_ in the atmosphere, so several measures have been taken to enhance some natural carbon sink processes to enhance the carbon sink to maintain the carbon balance (Table 1).

**Table 1:** Comparison of different land- or ocean-based technologies.

Type	Earth system	Mechanism	Measure	Advantage	Disadvantage
Afforestation and reforestation	Land	Photosynthesis	Mixed plantation; Plantation management	Ecological and economic benefits	Long growth cycle; Large land occupation
Soil carbon sequestration	Land	Photosynthesis; Microbial CO_2_ fixation	Fertilization and organic amendments; No-tillage systems; Crop rotation, Cover cropping	Large carbon sink capacity; Improvement of land quality	Availability of suitable land
Biochar	Land	Photosynthesis; Physicochemical process	High-temperature pyrolysis and hydrothermal carbonization of biomass or other organic matter	Improving soil fertility; Reducing the use of chemical fertilizer; High stability	High-temperature preparation process
Bioenergy with carbon capture and storage	Land	Photosynthesis; Physicochemical process	Pyrolysis, gasification, fermentation, or combustion of biomass or other organic matter to produce bioenergy; Carbon capture technologies	Renewable, low pollution; A wide range of raw material sources	High cost; Land occupation; Part of the bioenergy is made from food crops
Enhanced weathering	Land	Physicochemical process	Addition of carbonate or silicate	Increasing the essential nutrients; Mitigation of ocean acidification	High cost; Increasing soil pH
Ocean alkalinization	Ocean	Physicochemical process	Addition of alkaline minerals	Mitigation of ocean acidification	Increasing ocean pH; Affecting marine communities
Ocean fertilization	Ocean	Photosynthesis	Addition of iron, nitrogen, or phosphorus	Increasing productivity of marine life	Red tides; Production of other toxic acids; Lower oxygen levels in the ocean

### 2.1 Afforestation and reforestation

Forests play an essential role in global sustainability because of their ability to combat global warming, protect biodiversity, provide food, and improve environmental quality [20,21]. However, with economic development and population growth, the increasing demand for agricultural products results in the demand for agricultural land, and forests have faced the problem of over-exploitation. About 420 Mha of worldwide forests have been degraded and deforested since 1990, and approximately 130 Mha of tropical and subtropical forest cover loss was estimated between 1900 and 2016 [[21], [22], [23]]. Deforestation and forest degradation are among the largest sources of anthropogenic emissions of greenhouse gases to the atmosphere, contributing to almost 20% of total anthropogenic carbon emission annually through the combustion and decomposition of forest residuals, second only to the fossil fuels that burn out [24,25]. Therefore, afforestation and reforestation are considered as one of the most common approaches to combat greenhouse gas emissions. Afforestation refers to the establishment of trees in places where there has been no forest cover recently (at least 50 years), while reforestation refers to the re-establishment of forested lands that were recently deforested [24,[26], [27], [28]]. Since forests usually grow slowly and inconspicuously, carbon density changes in forest biomass and soil are often not intuitively perceptible by humans, leading to the underestimation of the forests’ contribution to terrestrial carbon sinks [29]. But tropical forests, as important carbon sources and sinks, play a huge role in stabilizing the climate. Tropical forests cover 96% of the world’s tree species and account for about 33% of the net terrestrial primary productivity, storing 25% of the global carbon [30]. The “Reducing Emissions from Deforestation and Degradation (REDD)” program, proposed at the United Nations Framework Convention on Climate Change (UNFCCC) in 2007, was seen as a cost-effective approach to mitigating global climate change for developing countries [24,31,32].

Good forest management can significantly increase the forest carbon sink and achieve twice the result with half the effort. Many factors affect the ability of forests to sequester CO_2_ from the atmosphere, such as vegetation species, climate conditions, soil conditions, and mixed plantation types [[33], [34], [35]]. In tropical rainforests, the rate of biological nitrogen fixation is high, and nitrogen does not become a limiting element for plant growth. However, this is different in tropical plantations with nutrient-poor sandy soils, where regular nitrogen supplementation is required. In order to save manpower and material resources, the introduction of nitrogen-fixation tree species may be an optimal choice to maintain tree growth, such as *Eucalyptus* spp [36]. In addition, mixed plantations can acquire and retain more carbon than monocultures [34,37]. With a meta-analysis of the relevant data, Xiang et al. found that mixed plantations could have a 12% higher carbon capture capacity than monocultures when the mixing ratio is less than 55% [34]. A reasonable portfolio of vegetation species should be adopted in the process of afforestation and reforestation to maximize the potential of forests to sequester greenhouse gases.

### 2.2 Soil carbon sequestration

Soils are a critical component of the global cycle and the largest reservoirs of the global terrestrial carbon stocks [27,38]. Soil carbon sequestration refers to the storage of captured atmospheric CO_2_ with the pedosphere in a manner that could increase its mean residence time and minimize the possibility of re-emissions [39,40]. The emission of CO_2_ can be offset by increasing soil organic carbon and reallocating atmospheric CO_2_ to soil carbon pools [41]. Significant efforts have been devoted to increasing soil carbon sequestration, including fertilization and organic amendments, no-tillage systems, crop rotation, and cover cropping [40,42,43]. The effectiveness of these practices is closely related to the activities among the microbes [44]. For example, fertilization is an essential part of agriculture, and long-term variations in pH values, soil nutrients, and soil microbial community structures caused by different fertilization modes may ultimately affect the production and decomposition of soil organic carbon [40,45]. A long-term experiment in an alpine meadow on the Tibetan Plateau was conducted by Yuan et al. [46], which revealed that the addition of phosphorus promotes the generation of particulate organic carbon due to the suppressive decomposition by lower fungal biomass. In contrast, the particulate organic carbon was barely changed by nitrogen addition, likely improving the ability of soil microbes to decompose biomass inputs. Although it reduced the limit of soil nitrogen to plant growth, the carbon added to the soil via plant biomass was rapidly broken down [46]. Therefore, understanding the soil microbial communities, their key role in carbon cycling, and their interactions with the soils are necessary to develop and adopt effective agricultural measures.

### 2.3 Biochar

Biochar is a solid substance with a high carbon content, which can be formed by pyrolysis under a high temperature (generally below 700 °C) with an oxygen-limited condition. Biochar has a long history and is rooted in the practice of the indigenous soil in the Amazon region called Terra Preta de Índio (also known as Amazon Dark Earths), which has nothing to do with modern environmental governance and was created by the indigenous inhabitants of the Amazon [47,48].

**Fig. 2:**
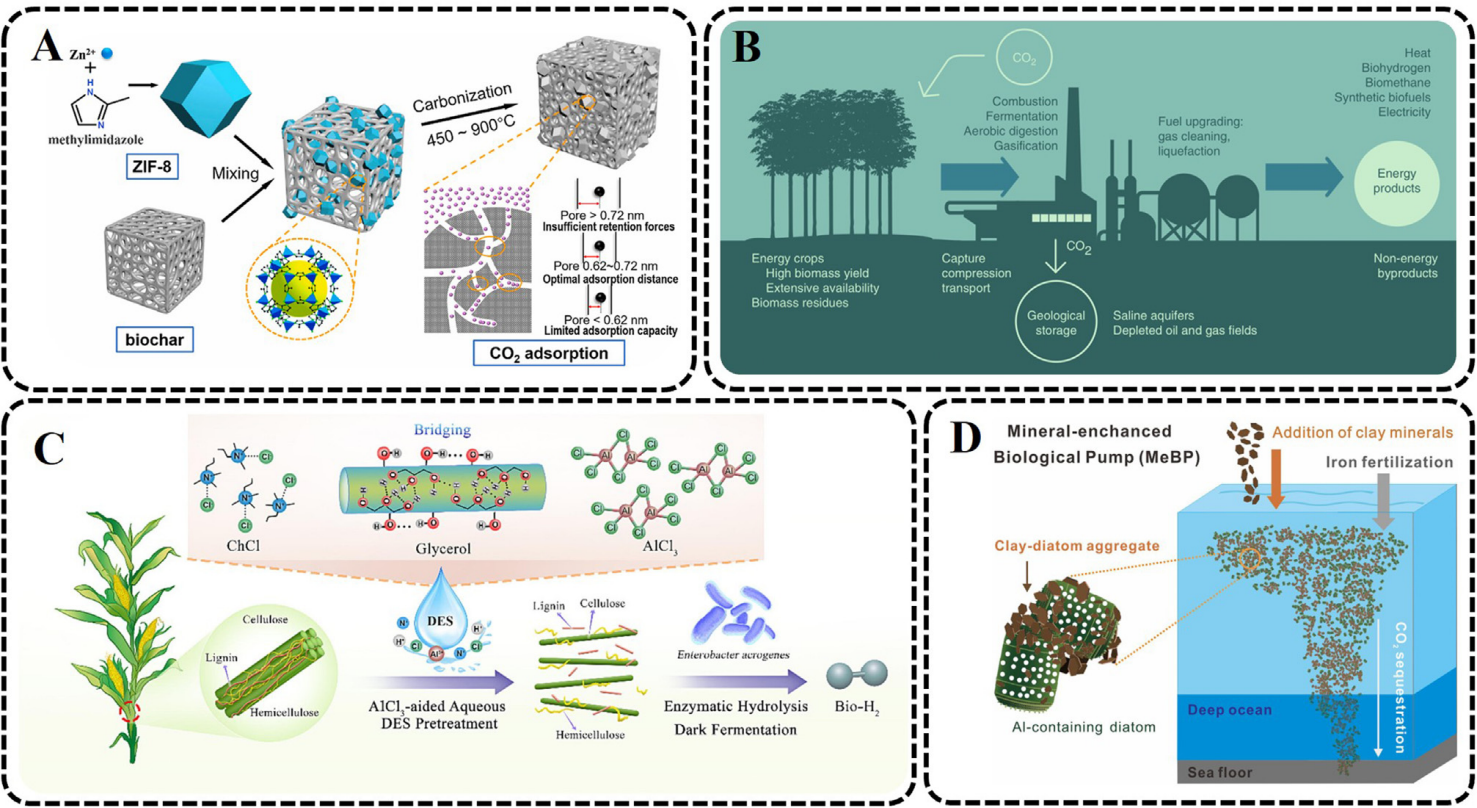
Examples of land- or ocean-based technologies for carbon neutrality. (A) Schematic diagram of nitrogen-doped biocarbon preparation and CO_2_ adsorption mechanism (Reprinted from ref. [57], Copyright (2022), with permission from Elsevier). (B) Schematic illustration of bioenergy with carbon capture and storage (Reprinted from ref. [61], Copyright (2014), with permission from Springer Nature). (C) Schematic diagram of enhanced enzymatic hydrolysis and biohydrogen production of corn straw with an AlCl_3_-aided aqueous deep eutectic solvent (Reprinted from ref. [65], Copyright (2022), with permission from Elsevier). (D) Schematic representation of the idealized mechanisms of the mineral-enhanced biological pump (Reprinted from ref. [81], Copyright (2021), with permission from Elsevier). ZIF-8, zeolitic imidazolate framework-8; ChCl, choline chloride; DES, deep eutectic solvent; Bio-H_2_, biohydrogen.

The organic feedstock for soil amendment could form carbon-rich charcoal substances to mitigate climate change [49,50]. Unlike other agricultural organic materials, biochar with a high proportion of recalcitrant organic carbon and a highly condensed aromatic structure has great carbon sequestration potential, which can lock most of the biomass carbon without releasing it into the atmosphere, reducing carbon emissions in the soil [[48], [49], [50], [51]]. Biochar can be prepared by many easily available biomass and waste materials as feedstock, for example, rich straw, wheat straw, sawdust, sugar cane bagasse, bamboo, hardwood, and sewage sludge [[52], [53], [54], [55]]. The influence of different biochar on the potential to store carbon in soil was evaluated by Tarin and coworkers [52]. Three different types of biochar at varying concentrations produced by bamboo, hardwood, and rice strew, respectively, were mixed with the soil in outdoor pots. The experimental result indicated that the cumulative CO_2_ emissions were greatly affected by the biochar level, and there was a significant interaction between biochar type and the level of cumulative CO_2_ emissions. Among them, the biochars prepared from hardwood and rice strew had a solid ability to isolate the carbon. Feedstock type and production conditions have a significant impact on the biochar adsorption of CO_2_. At the same time, there are many studies to effectively improve the adsorption characteristics of biochar through modification [56,57]. A clean modification method for activation-free synthetic nitrogen-doped biochar, which is obtained by pyrolysis after mechanical mixing of biochar particles and ZIF-8 particles, was developed by Zhang et al. [57], as shown in Fig. 2A. For the modified biochar, its surface area increased substantially from 3.0 m^2^/g to 989.3 m^2^/g, and its CO_2_ adsorption capacity increased from 0.52 mmol/g to 2.43 mmol/g. The improvement is mainly due to the fact that oxygen groups promote the CO_2_ affinity of nitrogen groups, and they have a significant synergistic effect on the adsorption of CO_2_. Moreover, the pore size of 0.62–0.72 nm plays a major role in the CO_2_ adsorption on the modified biochar. In practical application, appropriate biomass raw materials and preparation conditions should be selected, and nitrogen-containing and oxygen-containing groups should be modified to maximize the potential of biochar to mitigate climate change.

### 2.4 Bioenergy with carbon capture and storage

Bioenergy with carbon capture and storage (BECCS), owing to the advantage of generating energy and sequestrating atmosphere carbon, has been considered an attractive and pivotal carbon and storage method [58,59]. Biomass can be converted into a variety of bioenergies through pyrolysis, gasification, fermentation, or combustion, which is accompanied by the production of CO_2_ streams with different flows and concentrations. The resulting CO_2_ is captured and stored in geological formations, as indicated in Fig. 2B [60,61]. Biomass used for BECCS comes from a wide range of sources, such as crops, forestry residues, municipal organic waste, solid waste, landfill gas, marine microorganisms, and algae [59,62,63]. Most integrated assessment models suggest that BECCS is a key approach to reducing CO_2_ concentrations and avoiding catastrophic climate change [59]. In contrast, the carbon capture and storage of fossil fuels is pseudo net zero emission, because the carbon captured from fossil fuels is originally from the geosphere. BECCS temporarily transfers the carbon in the atmosphere to the biosphere through plant photosynthesis, which will then be permanently stored in the geosphere. Without capture and storage, the utilization of bioenergy is, at best, carbon neutral. BECCS could be applied to various industrial sectors such as combined heat and power plants, combustion biomass power plants, pulp industry, ethanol fermentation, and biomass gasification [61]. BECCS is essentially the combination of fuel conversion technologies and carbon capture technologies, and here is only for the current bioenergy production technologies, with a section on the carbon capture technologies later.

BECCS may be one of the important ways to achieve sustainable development. The techno-economic and environmental feasibility of sustainable aviation fuels produced through forestry residues-derived syngas via Fischer-Tropsch (FT) technology was studied by Michaga et al. [64]. This method can produce 1.91 tonnes of jet fuel from 20 tonnes of dry forest residues per hour, while 11.26 tonnes of CO_2_ are stored permanently. More importantly, the minimum selling price of sustainable aviation fuel prepared is only a little higher compared with traditional fuel, which has great potential for large-scale promotion. In addition, improving the efficiency of biomass use is also critical because biomass production is limited by land. Chen et al. developed an AlCl_3_-aided aqueous deep eutectic solvent (ChCl-glycerol) to improve enzymatic hydrolysis rate and biohydrogen production from corn straw (Fig. 2C) [65]. Lignocellulosic biomass, which is widespread in agricultural wastes, is composed of lignin, cellulose, and hemicellulose. The addition of AlCl_3_ promoted the dissociation and removal of hemicellulose and lignin, making enzymes and microorganisms approach the fermentable substrate and improve substrate utilization and hydrogen production efficiency. This may be mainly due to the build of the chlorine ions-glycerol-metal cation system, which increases the reactivity of the deep eutectic solvent. In addition, the production of biomass should be maximized through reasonable planting.

### 2.5 Enhanced weathering

Weathering releases alkaline components from carbonate or silicate, mainly as HCO_3_^−^, the main form of dissolved inorganic carbon in freshwaters, by consuming CO_2_ that comes from the atmosphere, thus sequestering CO_2_ in terrestrial waters or the ocean [66]. Nature weathering is a significant but very slow carbon cycle process, which can consume about 0.25 Pg C/yr of atmospheric CO_2_, accounting for approximately 3% of fossil fuel emissions [67]. Enhanced weathering is facilitating this process through human intervention, e.g., the distribution of pulverized rocks rich in alkaline minerals in farmland and woodland to further offset anthropogenic carbon emissions, including crushed calcium- and magnesium-rich carbonate and silicate rocks [[66], [67], [68], [69]]. It is a chemical storage pathway based on the reaction of CO_2_ with alkaline earth metal oxide-rich minerals [70]. Although silicates containing calcium and magnesium may not be as reactive as hydroxide minerals, their abundance makes them ideal candidates for enhanced weathering studies [71]. Agricultural land can provide a large area for the application of silicates to enhanced weathering. Hence, it is not only necessary to study the effect of certain alkaline minerals on carbon sequestration, but also to consider the effect of adding minerals on soil quality and plant yield, especially in highly weathered acidic and nutrient-deficient soil [72]. Silicon, a non-essential plant micronutrient, helps plants to increase yield and improve the tensile strength and thermal stability of natural fibers [73].

Haque et al. investigated the influence of wollastonite (CaSiO_3_) enhanced weathering on plants in soil [71]. It was found that soil amendment with wollastonite can promote the growth of plants (beans and corn). Meanwhile, the amount of CO_2_ sequestered by the wollastonite-amended soil was nine times higher than that without wollastonite amendment. Jariwala et al. found that improving different types of wollastonite skarn in the soil could make agricultural and horticultural plants grow better and sequestrate inorganic carbon [72]. In addition, a strategy of coating the mineral onto the fertilizer was developed to avoid the application of minerals alone and improve the plant uptake of the nutrient. Enhanced weathering is not limited to the addition of individual minerals to increase carbon capture, but requires an understanding of the effects of enhanced weathered minerals on soil nutrients and plant growth to achieve enhanced weathering and ecological effects synergy.

### 2.6 Ocean alkalinization

The ocean is the second largest reservoir of carbon, after the solid Earth, far beyond the atmospheric and biological reservoirs, and plays a central role in regulating Earth’s climate [74]. Due to its large volume and wide range, the ocean constantly exchanges CO_2_ with the atmosphere rapidly through the sea and air interface, thus controlling CO_2_ concentration in the atmosphere. Ocean alkalinization, also known as ocean liming, is a method of improving the CO_2_ capacity of the ocean by adding alkaline substances such as calcium hydroxide to increase the pH of the ocean ecosystem and simultaneously slow down the speed of the ocean acidification [3,19,75]. At present, ocean alkalization still needs laboratory and field experiments to determine the consequences of application and its optimal deployment. One key issue to consider is the stability of alkalinity added to seawater [76,77]. Calcium carbonate minerals in seawater are already oversaturated, and the increase in total alkalinity and the corresponding shift in carbonate chemistry to higher carbonate ion concentrations may lead to a further increase in supersaturation and possible solid carbonate precipitation. Precipitation of carbonate minerals consumes alkalinity and increases dissolved CO_2_ in seawater, thus reducing the efficiency of CO_2_ removal by ocean alkalinization.

### 2.7 Ocean fertilization

Ocean fertilization allows ocean phytoplankton to proliferate rapidly by adding limiting nutrients like iron, nitrogen, or phosphate, which can absorb CO_2_ from the atmosphere through the enhancement of photosynthesis, with the expectation that carbon would sink with dead biological matter and be stored for a long time [26,63,78]. Despite the abundance of macronutrients in many open ocean regions, the phytoplankton biomass is limited, mainly due to the limited micronutrient (iron). Additionally, the chemical composition (by atom) of typical algal cells is 106 carbon : 16 nitrogen : 1 phosphorus : 0.0001 iron [79]. Therefore, ocean iron fertilization is a cost-effective option.

Emerson has developed a new method of ocean iron fertilization based on biogenic iron dust [80]. A poorly crystalline Fe-oxides, which could be dropped from high altitude by aircraft and dispersed into the ocean by wind, were produced by chemosynthetic iron-oxidizing bacteria. This method, based on biogenic iron oxides, overcomes the disadvantage of low bioavailability of phytoplankton to refractory crystalline iron oxide minerals. Furthermore, the iron powder has a long enough residence time in the photic area to increase the possibility of contact time with phytoplankton. Moreover, Yuan et al. found that transporting carbon to the deep ocean after ocean fertilization was not strengthened, which could lead to another carbon release (Fig. 2D) [81]. A biological carbon pump enhancement strategy is adopted to agglomerate with diatomaceous biogenic silica through clay minerals, while the aluminum of clay minerals can inhibit diatomaceous biogenic silica dissolution, benefiting the increase in diatomaceous biogenic silica settlement and reducing organic carbon loss. In the ocean fertilization process, the specific conditions of the ocean need to be considered so that the added nutrients can achieve maximum carbon sequestration efficiency. Meanwhile, attention should also be paid to whether the organic carbon captured by ocean phytoplankton can be permanently enclosed in the deep ocean.

## 3 Carbon capture technologies

Negative carbon emission technologies based on land and ocean are mainly intervened through human activities to improve the natural carbon sink capacity. However, these technologies are limited by land and ocean, and the natural carbon sink capacities are not unlimited as well. If human intervention exceeds the self-regulation capacity of the ecosystem, it may have an irreversible impact on the ecosystem and human health. To achieve ambitious climate goals, carbon capture technologies with greater potential and cost-effectiveness may flourish shortly.

In carbon capture and storage technology, CO_2_, generated during the combustion of fossil fuels and other processes, is collected and captured through carbon capture facilities before emission. The captured CO_2_ is compressed to form liquid CO_2_, which facilitates CO_2_ transport and storage, before being piped into underground storage facilities [82,83]. More than 90% of CO_2_ generated from power plants can be removed by such technology [83]. In this review, four carbon capture technologies are mainly summarized and compared in Table 2.

**Table 2:** Comparison of different CO_2_ capture technologies.

Type	Introduction	Advantage	Disadvantage	Application
CO_2_ captured by sorbents	The physical or chemical properties of sorbents are used to adsorb CO_2_	Convenient application; Rapid response; Controllable adsorption capacity	High use cost; Difficult to reuse the sorbent; Possible environmental impacts; Limited operation time	Industrial application
CO_2_ captured by membranes	Using the difference in permeability of different components, the purpose of separation can be achieved through a membrane	High separation accuracy; Fast separation speed; Excellent durability	High demand for equipment; Periodic replacement of membrane	Industrial application
CO_2_ captured by electrochemical separation	The adsorption and desorption of CO_2_ were realized by electrochemical means using electrolytes as a mediator	Availability of liquid products; High separation accuracy	High demand for equipment; Energy consumption is higher than other methods	Industrial; Research experiment
CO_2_ captured by algae cultivation	Algae capture CO_2_ through photosynthesis	Cost-effective; Sustainable; Applicable to a wide range of CO_2_ concentration; Produce value-added biomass	High footprint; Light-dependent; Operation and maintenance are complicated	Industrial; Research experiment

### 3.1 CO_2_ captured by sorbents

The technology of carbon dioxide capture by sorbents is one of the most commonly used technologies, which can be divided into liquid sorbents and solid sorbents according to the state of the adsorbents.

#### 3.1.1 Liquid sorbents

**Fig. 3:**
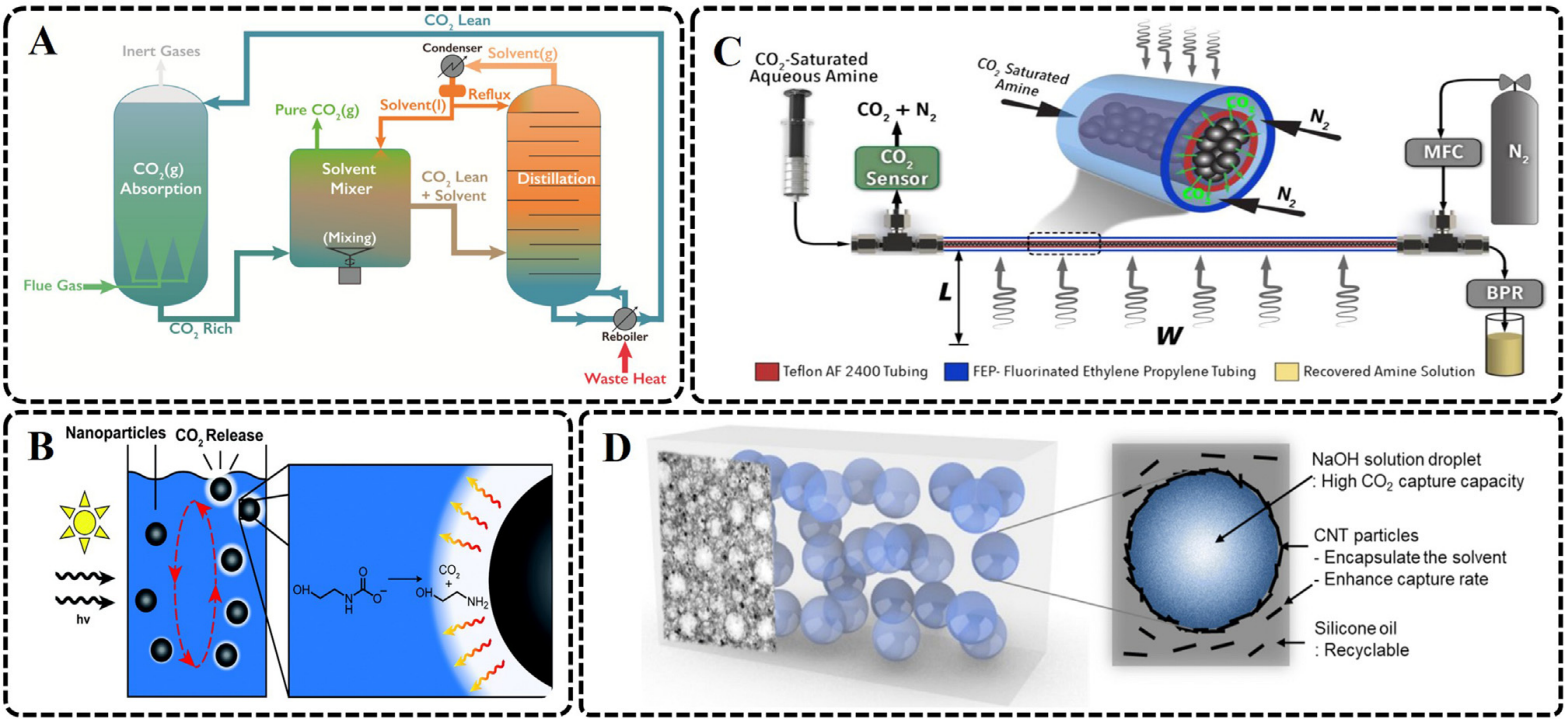
Example of CO_2_ capture by liquid sorbents. (A) Schematic of the solvent addition ammonia/carbon dioxide carbon capture process (Reprinted with permission from ref. [86]. Copyright (2016) American Chemical Society). (B) Schematic diagram of carbon black nanoparticle CO_2_ regeneration (Used with permission of Royal Society of Chemistry, from ref. [87], permission conveyed through Copyright Clearance Center, Inc.). (C) Schematic illustration of the developed flow chemistry platform utilizing a tube-in-tube packed-bed microreactor for continuous solar-enabled CO_2_ desorption from aqueous amines (Reprinted with permission from ref. [88]. Copyright (2021) American Chemical Society). (D) Concept illustration of the CO_2_ capture material based on the Pickering emulsion (Used with permission of Royal Society of Chemistry, from ref. [89], permission conveyed through Copyright Clearance Center, Inc.). *hv*, light energy; MFC, mass flow controller; *W*, Power of light source; *L*, distance; CNT, carbon nanotube.

The CO_2_ in the flue gas can be separated by a liquid sorbent, and then the used absorbent can be regenerated by heating, decompression, or stripping, while the CO_2_ released by the absorbent is stored. Due to the characteristics of cheap and easy access, liquid absorbers have gained wide popularity and are widely used in the industrial field, which is also one of the advanced technologies available today for negative carbon emission [84]. Amine scrubbing is identified as one of the earliest and most widely used technology to capture CO_2_ from flue gas, which also has a commercial application history of more than 90 years [85]. Unfortunately, one prominent drawback of the traditional CO_2_ absorption technologies is highly energy intensive and requires high-temperature heat sources and a large number of energy costs, which are mainly reflected in the re-release of carbon dioxide and the regeneration of absorbers. It is, therefore, necessary to improve the liquid absorption process to reduce the fossil fuel energy demand for CO_2_ regeneration [[86], [87], [88]]. Novek et al. have adopted a carbon capture technology that completely uses ample low-temperature waste heat from the thermal power industry as an energy source, which has not been fully utilized in some low-grade power plants due to thermodynamic limitations on the conversion of low-temperature heat into electricity (Fig. 3A) [86]. An aqueous ammonia solution containing a small amount of CO_2_ was used to absorb CO_2_ in the flue gas to form a CO_2_-rich solution in the technology. Adding water-soluble organic solvent (acetone, acetaldehyde, or dimethoxymethane) to the absorption solution produced high-purity CO_2_, and then the organic solvent was distilled by low-temperature waste heat. The regenerated aqueous ammonia solution and the organic solvent were further recycled. The low cost, widely available reagents, and running under room temperature and atmospheric pressure conditions make this strategy more competitive than other methods.

In addition, renewable energy is also a good alternative method, such as solar energy. Nguyen et al. reported the regeneration of CO_2_ from a carbon capture fluid (aqueous monoethanolamine solution or a CO_2_-binding organic liquid) containing nanoparticles, which could absorb actinic light to produce a photothermal effect (Fig. 3B) [87]. Carbon black nanoparticles were adopted as strong absorbers of light. And in the presence of sunlight, the photo-thermal effect generated the gas film with a lower thermal conductivity around carbon black nanoparticles, which could insulate the nanoparticle surface to raise its surface temperature. As the film gets larger and larger, carbon black nanoparticles rise closer to the surface of the solution, releasing CO_2_ before the nanoparticles return to the solution, and the process repeats. High CO_2_ regeneration efficiencies were obtained by increasing the nanoparticle concentrations and the initial solution temperatures. These photothermal nanoparticles used sunlight, instead of fossil energy, to quickly reach the temperature to regenerate the capture fluid, and could incorporate CO_2_ capture fluids at low loading levels without compromising the chemical absorption performance of the absorbent. Although the strategy of adding photothermal nanoparticles has achieved the desired effect, there are some technological problems, including the weakening of light penetration, the settlement of the particles, and the damage to the relevant equipment. Recently, Campbell et al. employed a microscale fluidic strategy that can use solar energy to drive continuous desorption while recovering CO_2_ captured from aqueous amine solutions (Fig. 3C) [88]. Light-absorbing graphite-titania composite microparticles were prepared as fillers of the optically transparent flow reactor for efficient solar desorption of CO_2_ from saturated aqueous amine absorbents. The flow reactor using graphite-titania composite microparticles has the highest desorption efficiency, the lowest pressure drop, and, therefore, the lowest cost compared with other light-absorbing materials, including titania and carbon black. Moreover, the developed flow chemistry platform has excellent stability with dynamic solar irradiance.

At present, many liquid absorbents have been developed for industrial high-concentration CO_2_ emission sources, such as coal or oil-fired boiler, natural gas turbine exhaust, and blast furnace gas cement kiln off-gas, among others. Unfortunately, these absorbents may not be suitable for an indoor environment with low concentrations at the ppm level. For example, some common liquid absorbents (such as monoethanolamine, methanol, and methyldiethanolamine) may face several challenges, including toxicity, volatileness, and corrosiveness, as well as performance degradation of absorbents. Strong alkaline NaOH is an inexpensive and nonvolatile absorbent that can react with CO_2_. In order to ensure the safety of indoor CO_2_ capture, NaOH solution should be in a stationary fluid state. However, NaHCO_3_ crystallization would be generated by the reaction of NaOH and CO_2_ at the gas-liquid interface, which could hinder the carbon capture process. A recyclable carbon nanotube/silicone oil emulsion with NaOH aqueous solution was adopted to overcome the difficulty by Lee et al. (Fig. 3D) [89]. Lipophilic-modified carbon nanoparticles have high dispersion stability in high-viscosity silicone oil, thus controlling the size of NaOH solution droplets and increasing the specific surface area and the rate of reaction with CO_2_. One gram of NaOH-based emulsion-type solvent can absorb 1.31 mmol of CO_2_. After CO_2_ capture, the absorbent can be easily separated from the aqueous and oil phases, and the oil phase can be reused.

#### 3.1.2 Solid sorbents

Solid adsorbents, compared with liquid absorbents, have the advantages of non-volatility, high concentration of CO_2_ output, and low energy consumption [90]. CO_2_ adsorption depends on the porous structures (pore sizes and pore geometries) and the electron density of the solid adsorbents. Generally, large surface areas and basic groups are more likely to adsorb more gas. According to the pore size, the solid adsorbents can be divided into microporous materials (<2 nm), mesoporous materials (2–50 nm), and macroporous materials (>50 nm) [91]. The adsorption of CO_2_ by porous materials mainly occurs in micropores, and the adsorption process can be analogous to a pore-filling process. The Lennard-Jones potentials from around the pore wall will stack, leading to higher adsorption enthalpy and thus an enhanced filling process. The factor affecting the enthalpy of adsorption is usually the size of the adsorbent micropore. When the pore size is similar to the kinetic diameter of the guest molecule CO_2_ (0.33 nm), it takes a very long time to fill all pores and reach the adsorption equilibrium [92]. On the contrary, if the size of the micropore is relatively large (more than 2–3 times the kinetic diameter), the increase in the adsorption enthalpy can be ignored, and correspondingly little CO_2_ enters automatically into the adsorption sites, especially at low concentrations [93]. Therefore, the bulk of efforts has been made to prepare outstanding adsorbents with suitable pore structures and chemical properties for selective adsorption of CO_2_, including carbon-based porous materials, zeolites and metal-organic structures, silica, activated carbon, and the constituents that demonstrate high affinity towards CO_2_ adsorption even at low concentrations.

**Fig. 4:**
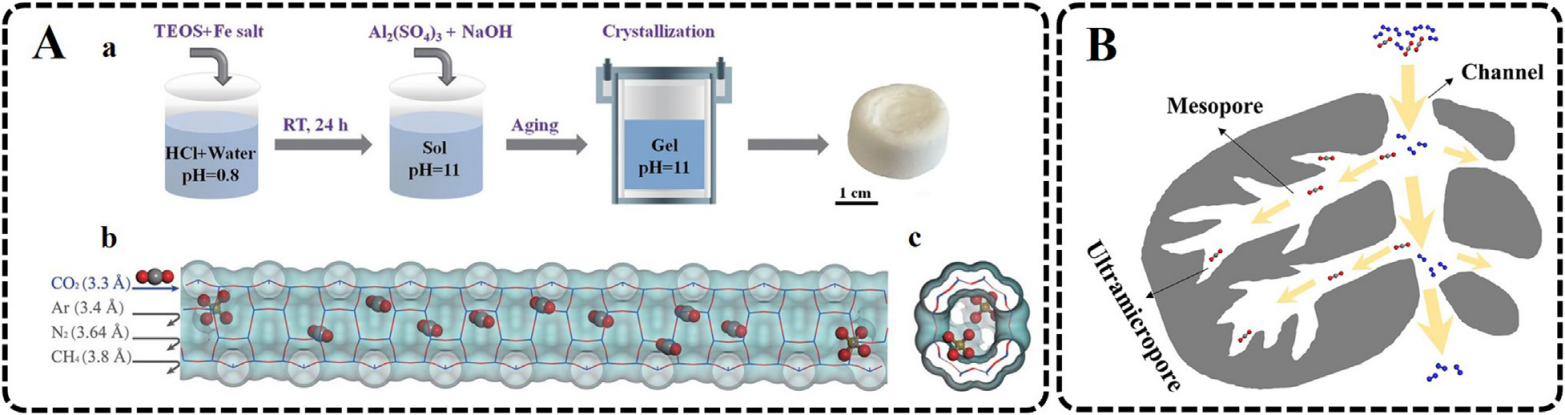
Examples of CO_2_ capture by solid sorbents. (A) Self-assembly of Fe-MOR monoliths. Shown are schematic illustrations of the synthetic procedure (a) and a side view (b) and top view (c) of precisely narrowed microchannels (kinetic diameter: 3.3 to 3.4 Å) by occupying isolated tetrahedral Fe species inside the 12-MR MOR microchannel. Blue indicates Si or Al, red is O, light brown is Fe, and gray is C (From ref. [94]. Reprinted with permission from AAAS). (B) Schematic diagram of enhanced CO_2_ adsorption capacity with well-defined hierarchical porous carbon with ultramicropore-mesopore interconnected pore architectures (Reprinted from ref. [93], Copyright (2022), with permission from Elsevier).

Zhou et al. prepared a self-assembled iron-containing mordenite zeolite monolith, which can serve as a molecular sieve for selective rapid adsorption of CO_2_ from mixed gas, by a simple and template-free hydrothermal method (Fig. 4A) [94]. The incorporation of iron ions in the mordenite framework was achieved through an acid co-hydrolysis route that enables slow co-condensation of iron and silica/aluminum precursors in the initial gelation stage, achieving precisely narrowed microchannels to obtain molecular sieving abilities. The pore characteristics of the material confer high capacity and high selectivity for CO_2_ adsorption, as well as excellent moisture resistance capability and stable recyclability. In addition to the uniform pore structure, the solid adsorbent materials can also be designed as a hierarchical pore architecture combining a certain number of mesopores and abundant ultramicropores. On the one hand, ultramicropores provide adsorption sites for CO_2_. On the other hand, the mesopores serve as transmission channels for gas molecules, which reduce the resistance to diffusion and allow CO_2_ to quickly reach the ultramicropores. Liu et al. reported that a well-defined hierarchical porous carbon with ultramicropore-mesopore interconnected pore architectures prepared by dual metal in-situ activation could significantly enhance CO_2_ adsorption capacity at low partial pressure (Fig. 4B) [93]. The porous carbon materials synthesized by this dual metal in-situ activation method can form a considerable number of mesopores without sacrificing the distribution of ultramicropores. The unique structures significantly improve the transmission efficiency of CO_2_ and the accessibility of ultramicropores, which can reach equilibrium in a short time. Moreover, the introduced heteroatoms could also help to improve the selectivity of CO_2_.

### 3.2 CO_2_ capture by membranes

Membrane separation for CO_2_ belongs to an emerging technology, mostly based on the differences in solubility, diffusivity, adsorption, and absorption abilities of different gases on different materials. When not necessary to obtain high-purity products, membrane separation is a more economical and flexible technology compared to other separation technology [84]. Membrane separation of CO_2_ is currently becoming increasingly attractive in recent years [85]. For example, significant research efforts have been made in CO_2_-philic membranes prepared from poly(ethylene oxide) (PEO) and its derivatives, driven by their strong dipole-quadrupole interaction with CO_2_ molecules [95]. However, the performance of membrane-based gas separation technology is still restricted by the trade-off between selectivity and permeability expressed typically by Robeson’s “upper bound” [96].

Mixed matrix membranes, first reported in 1970, are usually composed of a processable polymer matrix and dispersed inorganic particles, including zeolite, carbon molecular sieve, carbon nanotubes, silica, graphene, or metal/nonmetal nano-sized particles. The disadvantage regarding the selectivity and permeability trade-off is overcome by the mixed matrix membrane materials, combining the excellent gas separation properties of inorganic materials and the outstanding mechanical properties and cost advantages of polymers [97]. While inorganic fillers provide a standout permeation pathway for smaller gas molecules, mixed matrix membranes face uncertain compatibility between fillers and polymers as the proportion of inorganic fillers increases, resulting in undesirable defects between the inorganic material and the organic matrix and uneven dispersion of fillers. A facile *in situ* bottom-up growth approach has been proposed for the preparation of mixed matrix membranes with considerable organic loadings and enhanced permeability and compatibility [98]. The precursors of metal-organic framework (MOF) are uniformly dispersed in a polymerization mixture for the polymer membranes. The mixture was placed at the melting temperature of the polymer for the purpose of rearranging the MOF precursor and enabling its uniform distribution in rubbery cross-linked poly(ethylene oxide)-based polymer membrane. The MOF nanocrystal content in the obtained hybrid membranes was up to 67.7 wt%, and the MOFs were densely and orderly distributed on the polymer support. Notably, compared with other studies related to the “bottom-up” strategy, the methodology employs a solvent-free approach to dissolve the MOF precursors in poly(ethylene oxide) containing monomers, which is beneficial to generating a well-ordered array of MOF precursors. The CO_2_/N_2_ selectivity and CO_2_ permeabilities were 38.5 and 1083.7 barrier, respectively, which were significantly higher than the performance of conventional polymer membranes. Alternatively, a method of polymer blending was used to avoid the uncertain compatibility between polymers and fillers. A one-step approach to reducing crystallinity and improving mechanical strength by the *in situ* polymerization of highly flexible polyethylene glycol was proposed by Zhu et al. [99]. Sufficient entanglement between short poly(ethylene oxide) chains and the polymer matrix made the loading of linear polyethylene glycol very high. The robust structure gives polymer membranes enhanced gas separation properties and toughness, demonstrating the great potential of industrial carbon capture and gas purification.

### 3.3 CO_2_ capture by electrochemical separation

Electrochemical capture of CO_2_ dates back to the late 1960s when molten carbonate electrolytes were used in manned spacecraft to control the concentration of CO_2_ in the cabin [100,101]. Electrochemical CO_2_ capture has widely received increasing attention for its outstanding advantages of non-degradable operation, high energy efficiency, and flexible operation for easier retrofits [102]. Electrochemical processes directly target molecules rather than the surrounding medium and thus have considerable energy-saving potential [103]. In addition, The electrochemical capture device can be retrofitted as a plug-and-play process with a flexible shape and small footprint, which does not require a source of heat or high pressures/vacuum to operate [104]. Current methods related to the electrochemical capture of CO_2_ could be to any scenario containing CO_2_, such as air, flue gas, and ocean, regardless of the concentration [100].

In addition, electrochemical reduction of organic redox compounds (e.g., quinones, thiols, and bipyridines) is used to generate nucleophiles that could bind to the electrophiles with sufficient Lewis acidity CO_2_ to selectively capture CO_2_ from the gas mixture [105]. Nevertheless, most methods are limited by the presence of oxygen, because the reduced carriers can react with O_2_, causing unproductive carrier oxidation and the production of superoxide (O_2_^·−^). O_2_^·−^ could react with the carrier, electrolyte, and solvent [106]. To overcome this shortcoming, Barlow et al. adopt a quinone redox carrier modified with common alcohol additives to obtain an anodic change of the reduction potential to improve the selectivity of electrochemical carbon capture [107]. The intermolecular hydrogen-bonding interactions produced by the addition of alcohol tune the properties of redox carriers into ideal ranges that cannot be achieved through traditional molecular functionalization, showing O_2_ stability. There are also electrochemically controlled pH swings to drive CO_2_ capture [108]. This approach takes advantage of the responsiveness of the thermodynamic equilibrium of CO_2_ to pH changes [100,109].

### 3.4 CO_2_ capture by algae cultivation

Biological CO_2_ sequestration is receiving increasing interest worldwide for its advantages in cost and scalability. Plants consume 100 Gt CO_2_ annually through photosynthesis [110]. Thereinto, the efficiency of CO_2_ captured by algae is 10–50 times higher than that by the embryophyte (i.e., green plants) due to their high growth rate [111]. Each kilogram of algal biomass consumed 1.48–1.98 kg CO_2_, with an average value of 1.81 kg CO_2_ [112]. Zhao et al. [113] have estimated that a cultivation area of 100,000 km^2^ can capture up to 2.35 Gt CO_2_, accounting for 5%–8% of global carbon emission. The outstanding ability to utilize CO_2_ makes algae the ideal organisms for carbon capture. Besides, most of the algae have evolved CO_2_-concentrating mechanisms (CCMs) to concentrate CO_2_ in the vicinity of Rubisco (the CO_2_-fixing enzyme), facilitating algae to capture low-concentration CO_2_ in the atmosphere [114]. Although the growth of many algae may be inhibited by 10% CO_2_, some strains (e.g., *Dunaliella tertiolecta*) can tolerate a high concentration, even 100% CO_2_ [115]. The wide applicable CO_2_ concentration range enabled algae to have enormous potential as carbon sinks of natural CO_2_ and absorbents of flue CO_2_. Furthermore, algal biomass is a sustainable source of the third generation of biofuels [116,117]. The CO_2_ emitted after biofuel usage can be re-captured into algal biomass, and thus recycled. Collectively, optimizing the cultivation strategy to accelerate algae growth is a promising way to achieve a carbon-neutral future.

The algal biofilm has recently emerged as an attractive alternative to traditional suspension cultivation techniques such as raceway ponds [118]. Due to the light-dependent nature of algae cultivation, the algae pond for suspension cultivation has to be shallow, which may occupy huge land resources. Comparatively, algal biofilm exploits the vertical space and minimizes the footprint by the delicate design of attaching surfaces [119]. Besides, algal biofilm consumed much less energy for biomass harvesting than suspension cultivation, saving 20%–30% of the overall capital cost [120]. A life cycle assessment between the raceway pond and the attached culture system finds that the algal biofilm produces 50% more biomass while consuming 55% less energy and 30% water. The enhanced biomass production and reduced energy consumption benefit the net increment of CO_2_ sequestration. Miyauchi et al. used an infrared analyzer to measure the CO_2_ amount captured by *Chlorella* biofilm [121]. They reported that the biofilm captured 11,000 t CO_2_/km^2^ and suggested optimizing the attaching surfaces (e.g., configuration and material) and supplying higher concentration CO_2_ to further increase the capture efficiency. With numerous novel biofilm reactors to be developed [118] and increasing efforts to optimize operating parameters (e.g., hydraulic retention time and light intensity) [122], adopting the algal biofilm for the full-scale practice of CO_2_ capture is on the horizon.

Bacteria co-exist in algal biofilm because the attaching surface is continuously exposed to the ambient environment, and an axenic environment is practically unaffordable. Bacteria can benefit algal growth and carbon recycling in symbiosis [111]. Typically, algae assimilate CO_2_ to produce organic substrates and oxygen for heterotrophic bacteria, and in turn, bacteria mineralize organic matters to supply inorganic nutrients and CO_2_ for algae [123]. The cross-feeding between algae and bacteria could significantly change the physicochemical conditions of the phycosphere [124]. This changes the way algae interact with inorganic nutrients, including CO_2_ [125]. Thus, CO_2_ released from bacteria is supposed to provide a high-concentration CO_2_ phycosphere that facilitates algae to uptake CO_2_. Besides, bacteria can also provide infochemicals (e.g., vitamin B12 and indole-3-acetic acid) to promote algal cell growth and division [124], which can indirectly increase CO_2_ capture. Nevertheless, bacteria are not always beneficial to the algae. For example, Pseudomonas protegens can substantially inhibit the growth and carbon fixation of *Chlamydomonas reinhardtii* [126]. Therefore, promoting the synergistic effect and reducing the antagonistic relationship between algae and bacteria are pivotal to constructing a symbiosis with high CO_2_ capture capacity.

## 4 CO_2_ utilization and conversion

After the CO_2_ is separated, it is usually compressed and transported to a suitable storage place through pipelines or ships [127,128]. Although the soil and ocean have a large storage capacity of CO_2_, such carbon storage technologies still face the potential risks of leakage and the accompanying second disasters. Meanwhile, as the cost of carbon capture and storage technologies increases, the strategy of CO_2_ utilization and conversion is considered an efficient, economical, and environmentally benign option that can not only solve the storage problem of captured CO_2_ but also realize its economic value [3,6,63].

### 4.1 CO_2_ utilization

CO_2_ has always been used in many industrial-scale applications, such as carbonated beverages, refrigerants, and fire extinguishers. In addition, liquid CO_2_ can replace organic solvents in biological medicine, polymer production, furniture manufacturing, and other fields, because the density of CO_2_ in the liquid state is comparable to that of other organic solvents with low viscosity and good wetting properties. And supercritical CO_2_ is also an excellent solvent that can be used in the cleaning, food, and electronics industries [6,129].

Besides the aforementioned applications, CO_2_-enhanced oil recovery is another promising carbon utilization technology that can not only increase oil production but also permanently store CO_2_ underground [130]. CO_2_ injection has been adopted to increase the recovery of oil reservoirs since the middle of the last century. The excellent dissolution properties enable CO_2_ to be mixed with the in-situ oil at a relatively small pressure (the minimum-miscibility pressure) and drag the oil out of the pores through the production of reduced interfacial tension, reduced viscosity, and oil swelling. The Department of Energy has always invested in the next generation CO_2_ enhanced oil recovery projects in the United States, and CO_2_ has been successfully stored in geological formations in many parts of the United States [131]. Regarding the CO_2_-enhanced oil recovery technology in conventional reservoirs matures, hundreds of CO_2_ projects have been implemented worldwide, producing more than barrels a day in the United States alone. In addition, the depleted oil and gas reservoirs have the necessary infrastructure and facilities, including injection wells, ground implementation, transporting pipelines, and the structural integrity containing fluids over a very long time, broadly recognized as ideal geological bodies for CO_2_ storage. Additional oil and gas are obtained when injecting CO_2_ into depleted oil as a replacement agent [4,132]. However, there are still some challenges in the co-optimizations of CO_2_ storage and oil recovery, such as CO_2_ override, viscosity fingering, and gravity segregation [133]. Liu et al. adopted a storage-driven CO_2_-enhanced oil recovery method that used dimethyl ether (DME) as an efficient agent to maximize oil recovery and achieve net-zero or even negative carbon emission [4]. The introduction of DME not only enhanced the solubility of CO_2_ *in situ* oil recovery but also suppressed the “escape” of lighter hydrocarbons from the crude oil, which is beneficial for the solubility capture of CO_2_ storage and sustainable oil recovery. It is noteworthy that the amount of emission from the combusting of the oil is less than the amount of sequestrated CO_2_ in storage-driven CO_2_-enhanced oil recovery. That means sequestrated CO_2_ includes both current emissions and past emissions.

### 4.2 CO_2_ conversion

CO_2_ conversion to value-added fuels and chemicals is deemed an effective method of much interest because it can help reduce excess CO_2_ emissions as well as the consumption of nonrenewable energy resources to alleviate the energy crisis. CO_2_, as an unavoidable product of the oxidation of organic molecules during biological respiration and the combustion of carbon-containing molecules, such as fossil fuels, is kinetically and thermodynamically stable and difficult to be chemically activated to react. Therefore, an excellent catalyst featured with the ability to promote CO_2_ reduction has long been sought after [134,135]. The CO_2_ catalytic reduction process is a complex multi-step reaction, which, according to the energy source, can be divided into photocatalytic reduction, electrocatalytic reduction, and thermocatalytic reduction (Table 3). In addition, some combined technologies have been proposed, for example, photoelectrocatalytic reduction and photothermal catalytic reduction. Each method has its advantages and limitations, but should not be considered competitors or substitutes [136]. CO_2_ reduction could produce various chemicals, including carbon monoxide (CO), methane (CH_4_), methanol (CH_3_OH), formate (HCOOH), and other longer-chain hydrocarbons [137]. In short, CO_2_ reduction conversion is to achieve negative carbon emissions under the action of catalysts through solar, electricity, or thermal energy, leading to the permanent removal of CO_2_ from the atmosphere. Unlike sustainable fuel production, the energy density carried by the transformed products is not particularly important and is often more focused on carbon density [138]. In addition, captured CO_2_ can usually be used as raw material for CO_2_ conversion through release, compression, transportation, storage, and other steps, which require high energy consumption and cost. Therefore, researchers are trying to integrate the processes of CO_2_ capture and conversion, that is, directly converting the captured CO_2_ into value-added products [139]. Moreover, many infrastructure departments have made efforts to reduce emissions and achieve sustainability, such as wastewater treatment plants, which are considered to be one of the largest sources of greenhouse gas emissions [140]. Reasonable wastewater treatment design can offset the greenhouse gas footprint of the industry and promote it to become an important contributor to global negative carbon emissions [141]. In addition, some studies reported co-conversion of CO_2_ with other air pollution gas, which improves the conversion efficiency [142,143].

**Table 3:** Comparison of different CO_2_ reduction technologies.

Type	Energy	Temperature	Advantage	Challenge
Photochemical CO_2_ reduction	Photon	Ambient temperature	Environment-friendly; Energy conservation; Simple operability.	Low photon efficiency; Low product selectivity and reaction rate; Expensive catalysts; Low stability of the catalyst.
Electrochemical CO_2_ reduction	Electricity	Ambient temperature	Environment-friendly; Mild operating conditions; Good product adjustability; Rich product types.	High overpotential; Low solubility of CO_2_ in aqueous solutions; Liquid product mixed with electrolyte; Low Faradaic efficiencies, current densities, and high energy consumption; Low stability of the catalyst.
Thermochemical CO_2_ reduction	Heat	500–1000 °C	High-value product; Efficient selectivity; Large scale.	High energy consumption, high-temperature, and high-pressure conditions.

#### 4.2.1 Photochemical CO_2_ reduction

In nature, green plants use solar energy for photosynthesis to balance the carbon/oxygen cycle, which provides the energy needed for life on earth and the basis of human survival. Over the past three decades, researchers have tried to imitate the exquisite process in nature. The purpose is to use solar energy as the sole energy source to convert atmospheric CO_2_ into value-added chemicals [144,145]. Because it does not require additional energy intake, artificial photosynthesis is considered to have great potential to make a significant contribution to our future energy supply [146].

Sunlight is mainly composed of ultraviolet (UV) light (λ < 400 nm), visible light (400 nm < λ < 800 nm), and infrared light (λ > 800 nm), which account for 4%, 53%, and 43% of the whole solar energy, respectively [147]. To achieve maximum solar energy utilization efficiency, photocatalysts that respond to visible light are developed. The catalysts can form the photogenerated electron and hole pairs under sunlight irradiation conditions, which migrate to the surface of catalysts to participate in reduction and oxidation processes, respectively [148]. Having more accessible active sites and absorbed CO_2_ concentration on the photocatalysts could be beneficial to accelerate the CO_2_ reduction process [149].

**Fig. 5:**
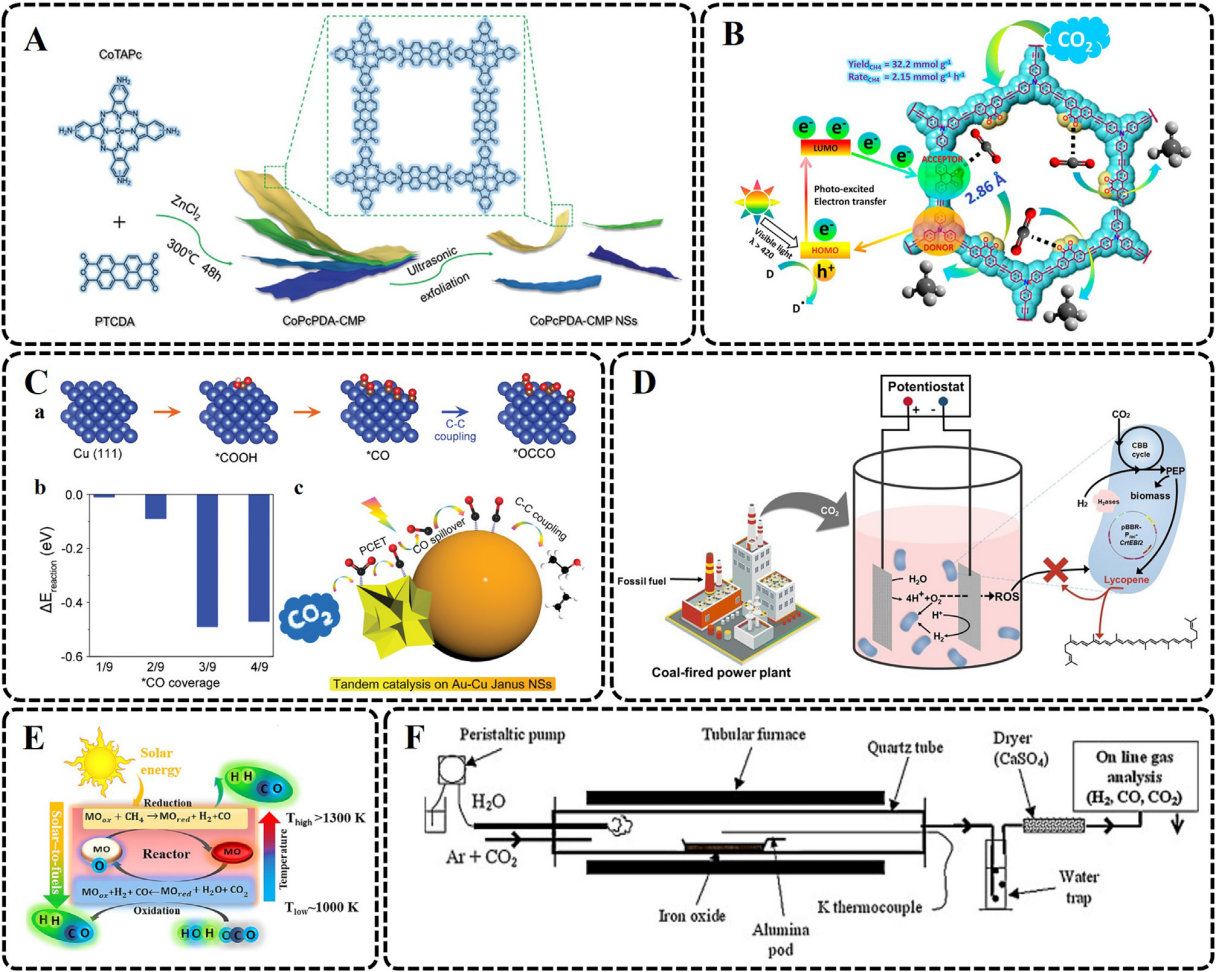
Examples of different CO_2_ reduction technologies. (A) Schematic illustration of CoPcPDA-CMP NSs synthesis (Reproduced with permission from ref. [157], Copyright (2022) John Wiley and Sons). (B) Schematic illustration of visible-light-driven CO_2_ reduction involving a donor–acceptor CMP with fast, selective, and efficient CH_4_ production (Reprinted with permission from ref. [158]. Copyright (2021) American Chemical Society). (C) DFT calculations. (a) Key reaction pathways for C_2+_ products formation via C–C coupling. Blue, copper; brown, carbon; red, oxygen; white, hydrogen. (b) Reaction energies (ΔE_reaction_) of the C–C coupling step at different ∗CO coverage on a 3 × 3 Cu(111) surface. (c) Scheme illustration for the tandem catalysis of CO_2_RR over Au-Cu Janus NSs (Reproduced with permission from ref. [178], Copyright (2022) John Wiley and Sons). (D) Schematic diagram of the MES system (Reprinted from ref. [191], Copyright (2022), with permission from Elsevier). (E) Solar thermochemical looping reforming process (Reprinted from ref. [198], Copyright (2020), with permission from Elsevier). (F) Tubular reactor for CO_2_ and H_2_O splitting from FeO particles (Reprinted from ref. [206], Copyright (2011), with permission from Elsevier). CoTAPc, tetraaminophthalocyanatocobalt(II); PTCDA, 3,4,9,10-perylenetetracarboxylic dianhydride; CoPcPDA-CMP NSs, 2D imide-based covalent organic polymer nanosheets; PCET, proton-coupled electron transfer; NSs, nanostructures; ROS, reactive oxygen species; PEP, phosphoenolpyruvate; pBBR-P_*lac*_-*CrtEBI2*, lycopene strain; H_2_ases, hydrogenases.

At present, extensive efforts have been made to develop various metal photocatalyst materials, such as metal oxides, metal sulfides, and MOFs [[150], [151], [152], [153], [154], [155], [156]], for efficient sunlight-driven CO_2_ reduction. 2D imide-based covalent organic polymer nanosheets (CoPcPDA-CMP NSs) were prepared using tetraaminophthalocyanatocobalt(II) (CoTAPc) and 3,4,9,10-perylenetetracarboxylic dianhydride (PTCDA) for photocatalytic CO_2_ conversion by Zhi et al. (Fig. 5A) [157]. The integrated cobalt phthalocyanine and 3,4,9,10-perylenetetracarboxylic diimide moieties realize the reduction of CO_2_ and the oxidation of H_2_O, respectively, leading to a Z-scheme charge transfer. Compared to other catalysts, the catalyst has excellent light absorption capacity, charge separation efficiency, and electronic conductivity on the photocatalytic of CO_2_. In addition, many studies are dedicated to developing metal-free photocatalysts (e.g., conjugated microporous polymers and covalent organic frameworks) [[158], [159], [160]]. Barman et al. used an electron donor (tris(4-ethynylphenyl)amine, TPA) and an acceptor (phenanthraquinone, PQ) to prepare a redox-active conjugated microporous polymer (CMP), TPA-PQ, as a metal-free catalyst, which was used to efficiently and selectively transform CO_2_ into CH_4_ by visible light as shown in Fig. 5B [158]. Due to the poor light utilization and rapid recombination of photogenerated carriers, the efficiency of photochemical CO_2_ reduction is still low [155,161]. Therefore, we need to construct more reasonable catalyst materials for photocatalytic CO_2_ conversion into high-value-added products to meet the requirements of actual industrial applications.

#### 4.2.2 Electrochemical CO_2_ reduction

From the perspective of technical and economic analysis, the electrochemical reduction of CO_2_ is considered a viable route for the production of carbon feedstocks for fuels and chemicals to achieve net-zero CO_2_ emission energy systems [162,163]. In electrocatalytic CO_2_ reduction reaction (eCO_2_RR), using water instead of molecular hydrogen can avoid high energy consumption from heating [164]. Combined with electricity from renewable sources, this method is expected to replace conventional petroleum-based processes for industrial implementation [165,166]. The technological viability of the process is contingent on the design and synthesis of highly efficient and selective catalysts for this reaction and their reaction mechanism.

In the complete electrolysis system, oxygen evolution generally occurs at the anode, while CO_2_ conversion occurs at the cathode [164,167]. The product distribution of CO_2_ electroreduction mainly depends on the cathode catalyst. And the highly efficient catalyst can greatly inhibit the process of hydrogen evolution reaction. Moreover, in order to meet the requirements of practical implementation, techno-economic analyses show that the Faradaic efficiency (FE) should be more than 90%, the partial current should be more than 200 mA/cm^2^, the energy conversion efficiency (EE) should be more than 60%, and the stable operation should exceed 1000 h, in the process of CO_2_ electroreduction generation of a single product [162,168].

CO_2_ reduction products have been mainly restricted to the C_1_ products and their derivatives in the last few decades [[169], [170], [171]]. For example, Our research group adopted a partial-carbonization strategy to prepare Cu single-atom catalysts for converting CO_2_ to CH_4_ [171]. And the overall endothermic energy of key intermediates was reduced by modifying the electronic structures of single-atom catalysts (SACs). The Faradaic efficiency of the electrochemical reduction to CH_4_ was as high as 78%, and the hydrogen evolution reaction was greatly inhibited with more than 99% of the reduction product being CH_4_. In most cases, reducing CO_2_ to C_2+_ hydrocarbons and oxygenates was more popular because of their better economic value, higher energy density, wider applications, and bigger contribution to decreasing net CO_2_ emission [172]. However, the current method of CO_2_ electroreduction to produce C_2_ products still fails to meet the thresholds necessary for industrial application, especially with regard to FE, EE, and stability [173]. The obstacles are associated with the fact that the fully oxidized CO_2_ requires large overpotentials to be thermodynamically activated. In addition, the multicarbon products generally involve the formation of the C–C bond via dimerization (in the form of CO–CO) or coupling (in the form of CO–COH) pathways, accompanied by multiple electron/proton transfers and multi-step oxidation/hydrogenation processes, which have significant energy barriers [174,175]. The HER at the cathode has close equilibrium potential to CO_2_ electroreduction, leading to reduced efficiency.

Metal catalysts are among the commonly used electrocatalysts for CO_2_RR, which can be divided into four categories depending on the binding ability of intermediates and products [146]. The first type is the CO-producing metal catalysts, including Au, Ag, and Zn. These catalysts have a strong affinity for ∗COOH intermediates to generate ∗CO, and the bond between ∗CO and the catalyst is relatively weak, resulting in desorption to release CO. The second type is HCOOH-producing metal catalysts, including Pb, Sn, Hg, and Bi, which have a low affinity for CO_2_^·−^. The third type is H_2_-producing metal catalysts, including Ni, Fe, and Pt, which have strong binding ability with ∗CO intermediates. The last type is hydrocarbons- and alcohol-producing Cu-based catalysts, which have negative adsorption energy for ∗CO intermediates and positive adsorption energy for ∗H. Cu is the most widely studied metal that exhibits a suitable binding capacity with the ∗CO intermediate, facilitating the hydrogenation reaction of the ∗CO and the C–C coupling reaction [[176], [177], [178]]. For example, Zheng et al. used a typical seeded growth strategy to prepare Au-Cu Janus nanostructures, which increased CO coverage on the Cu sites due to CO spillover from the adjacent Au sites (Fig. 5C) [178]. This tandem catalyst significantly reduces the C–C coupling reaction energy in favor of C_2+_ product formation. Alternatively, metal-free carbon-based materials for electrochemical CO_2_ reduction have received much attention due to their low cost and environmental friendliness [179,180].

Moreover, some studies are focusing on the combination of electrocatalysis and photocatalysis, namely photoelectrocatalysis, which combines the advantages of both [181]. On the one hand, the utilization of solar energy can significantly reduce the applied voltage, thus decreasing electricity consumption. On the other hand, the imposition of an external bias voltage can enhance the separation of electrons and holes to improve the efficiency of photocatalysis [182]. The external bias voltage may be provided by renewable electricity supplied by solar or wind energy, thus offering a sustainable solution for CO_2_ conversion. Photoelectrocatalysis, similar to photocatalysis, has three key steps, namely sunlight harvesting, charge separation/transport, and surface redox reaction [183]. To date, various semiconductor photocathodes, including p-Si, CdTe, p-InP, ZnTe, Cu_2_O, and p-NiO [[184], [185], [186], [187], [188]], have been studied for Photoelectrochemical CO_2_ reduction to CO, usually used in combination with molecular complexes or metal cocatalysts (including Ag, Au, and derivatives) to achieve selective CO production [189]. Photochemical reduction of CO_2_ is a high potential, but the expansion of the negative carbon emission technology portfolio in the current technology maturity situation still has a high risk [138].

Recently, microbial electrosynthesis (MES) systems, which combine the advantages of inorganic and microbial-catalyzed electrocatalysis, could synthesize multi-carbon organic chemicals using CO_2_ as a substrate and are considered a promising platform for CO_2_ reduction [[190], [191], [192]]. The MES systems consist of electrode materials, biocatalysts, reactors, and membranes, among which biocatalysts or microbial communities are considered to be important parts [190]. During the conversion process, electrochemical reactions provide the necessary redox equivalents (such as H_2_, electrons, and formate) to trigger certain biochemical reactions of microorganisms, making it possible to convert CO_2_ into complex molecules in one step [191,[193], [194], [195]]. Wu et al. developed an MES system for the one-step synthesis of lycopene utilizing CO_2_ as the sole carbon source (Fig. 5D) [191]. Lycopene can reduce the cytotoxic reactive oxygen species (ROS) produced by the electrochemical system and improve the compatibility between microbial and electrochemical catalysis. Moreover, the feasibility of lycopene production by the MES system was demonstrated by using the actual waste gas from the coal-fired power plant as feedstock without affecting the growth of bacteria. However, the yield of lycopene was not as high as that of other simple chemical products. It is necessary to further optimize the reactors and adopt biological methods to further modify the metabolic pathways for improving energy efficiency.

#### 4.2.3 Thermochemical CO_2_ reduction

In 1902, Sabatier proposed the feasibility of converting CO_2_ and H_2_ into CH_4_ [196,197]. At atmospheric pressure, the thermal catalytic reduction of CO_2_ is based on the reverse water-gas shift (RWGS) reaction and methanation reaction to produce CO and CH_4_. Although the thermal catalytic CO_2_ reduction has a high conversion efficiency compared with photocatalysis and electrocatalysis, the heat source is mainly from fossil fuels, which is not conducive to carbon neutralization. Therefore, most of the current research is combined with solar energy to reduce carbon emissions [[198], [199], [200]]. The photothermal effect is another way to use solar energy efficiently. Compared to conventional photocatalysis, which only harvests UV and part of visible light and wastes a lot of energy, photothermal catalysis utilizes the full wavelength range of the solar spectrum, especially the low-energy IR light, which not only performs electron/hole-engage redox reactions but also produces the photothermal effect caused by the photoexcitation of semiconducting materials to promote endothermic reactions [201,202]. There are currently three scenarios for photothermal catalysis: (1) The catalysts produce electron/hole pairs and heat, where photocatalysis and thermocatalysis are achieved, respectively; (2) Solar energy is only used as the heating source to provide photons, which can achieve photo-to-thermal conversion under irradiation on the catalyst. When reaching a certain temperature, photo-driven thermal catalysis occurs; (3) Solar energy is coupled to thermal energy resulting in a synergistic effect of photocatalysis and thermocatalysis [[203], [204], [205]]. A high-flux solar simulator filled with NiFe_2_O_4_@alumina for the thermochemical CO_2_ reduction to produce syngas was adopted by Lougou et al. (Fig. 5E) [198]. The NiFe_2_O_4_@Alumina porous medium in a solar reactor has good chemical energy-flux density, thermal conversion efficiency, and thermal-to-chemical energy conversion efficiency. Abanades et al. used an iron oxide redox pair to reduce CO_2_ through two-step solar thermal chemical looping to generate syngas (Fig. 5F) [206]. The first step was to oxidize iron(II, III) oxide to O_2_ and FeO in a high-temperature solar chemical reactor heated by concentrated solar energy. The second step was to convert CO_2_ and H_2_O into H_2_ and CO, while FeO decomposed into Fe_3_O_4_, which can be recycled to the first step indefinitely.

#### 4.2.4 Other CO_2_ conversion technologies

In addition to the three basic pathways of CO_2_ conversion, there are also numerous studies focusing on combining CO_2_ conversion with other applications. The significant advantage of this combination technique is that it can greatly reduce cost, improve efficiency, and promote synergy between different technologies.

##### 4.2.4.1 Integrate CO_2_ conversion with capture

Carbon capture technologies still have a high total cost, including capital costs and energy demand [207]. For specific carbon capture, the cost of CO_2_ from different sources also varies greatly. The cost of carbon capture in high-concentration CO_2_ streams generated in natural processing or ethanol production is USD 15–25/t CO_2_, while that in the low-concentration gas streams produced by cement production or power generation is USD 40–120/t CO_2_ [208]. Capturing CO_2_ from the air is the most expensive. For large-scale plants built today, the capture cost of CO_2_ per ton is between USD 125 and USD 335, which may decrease in the future as the technology matures [209].

While many CO_2_ conversion technologies have been reported in the previous literature, CO_2_ is not free. In fact, CO_2_ needs to be recovered from many sources of different dilutions, and the capture, desorption, compression, transportation, and storage of each CO_2_ molecule have operational costs. Therefore, the development of capture and conversion integration technology is an effective strategy to save costs and improve energy utilization, which can avoid the intermediate cumbersome steps from capture to conversion after energy-intensive capture and conversion coupling. The integration of capture and conversion can be divided into three categories according to the degree of direct coupling between the capture and conversion technologies, i.e., independent (Type-I), subsequent stage (Type-II), and fully integrated (Type-III) capture and conversion processes [210].

**Fig. 6:**
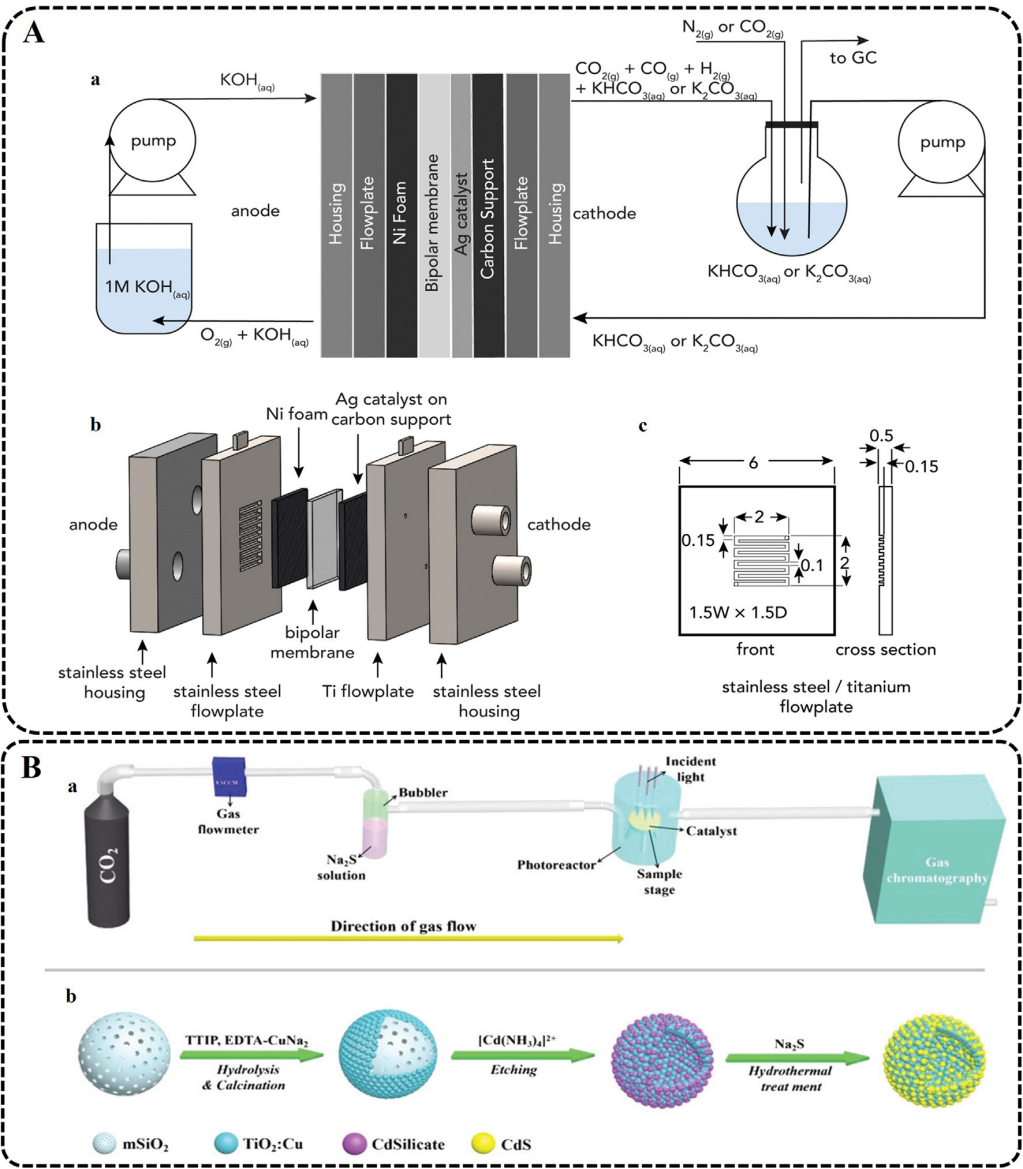
Examples of other CO_2_ conversion technologies. (A) Experimental setup of (a) electrochemical flow-cell experiment, (b) an expanded view of the flow cell, and (c) dimensions of the cathode and anode flow plates (Reprinted from ref. [214], Copyright (2019), with permission from Elsevier). (B) Schematic of (a) the online photocatalytic test system, and (b) the synthesis of CdS/TiO_2_:Cu hollow spheres (Reproduced with permission from ref.[142], Copyright (2022) John Wiley and Sons).TTIP, titanium isopropoxide; EDTA-CuNa_2_, ethylenediaminetetraacetic acid copper disodium salt hydrate.

The capture and conversion processes in Type-I occur independently with minimal correlation and strong flexibility. For example, the operating temperatures of the thermochemical release of CO_2_ and the solvent regeneration are not the same as that of the electrochemical reduction of CO_2_, which requires them to be completely separated [211,212]. However, because of this independence, the high-energy requirements and operating costs can not be significantly reduced in the operation process. Type-II involves the coupling of partial capture and conversion processes. Here, the reactants of the conversion process remain molecular CO_2_, but a flux match between the capture and the conversion process is required to achieve optimal system performance. For example, the electrochemical reduction of CO_2_ is combined with electrochemically mediated amine regeneration [103]. Type III, which bypasses conventional capture agents for releasing CO_2_ and directly electroreduction of CO_2_-loaded capture agents, is considered to be one of the most effective and promising approaches [210,213]. Li et al. built an electrochemical flow reactor to reduce the KHCO_3_ solution without providing a gaseous CO_2_ for the electrolyte, breaking the widespread perception that CO_2_ feed is necessary for the generation of CO (Fig. 6A) [214]. The bipolar membrane of the electrolyzer can transfer the generated H^+^ flux to the cathode chamber, where it converts HCO_3_^−^ and CO_3_^2−^ into catalytically active CO_2_. The Faraday efficiency of the KHCO_3_ system without CO_2_ feed to produce CO is comparable to that of similar systems with a CO_2_ feed solution. Regeneration of the capture medium and conversion of CO_2_ in the form of CO_2_ adducts occur simultaneously during this fully integrated CO_2_ capture and conversion process, significantly reducing the overall capture and conversion energy.

In addition to combining with electrocatalysis, a thermal catalytic reduction is also an alternative due to its high conversion efficiency. In response to the disadvantages of the high energy and cost required of carbon capture aqueous systems, Kothandaraman et al. have developed a combination of water-lean CO_2_ capture solvent and thermocatalytic conversion of CO_2_ to methane [215]. Moreover, the small amount of water in the solvent is also conducive to the shift of the reaction equilibrium toward the products, because most of the conversion reactions involve water as a by-product, such as conversion to methane, methanol, and ethanol. The high physical solubility of CO_2_ is another advantage of the water-lean capture solvents. More importantly, the heat released from hydrogenation during thermocatalytic reduction can also be used to offset the energy required for the regeneration of the captured solvent.

##### 4.2.4.2 Carbon conversion in wastewater treatment

With the growth of the population, the treatment of domestic sewage has become more and more important [[216], [217], [218], [219]]. CO_2_ and other greenhouse gases are released during the treatment process, making wastewater treatment plants one of the greenhouse gas emission major sources. In particular, with more and more stringent water-quality standards, CO_2_ emissions and sludge production will also increase, which stands on the opposite side of CO_2_ reduction targets [220].

Currently, most domestic wastewaters have adopted traditional or modified activated sludge processes, consuming huge energy and carbon footprints. The aforementioned algal biofilm (algal-bacterial symbiosis) has attracted increasing interest recently for its promising efficiency in nutrient (i.e., nitrogen and phosphorus) recovery [221]. Using wastewater to cultivate algae also reverses its high demand for nutrients into an advantage. More importantly, organic matter in wastewater could also be converted into algal biomass for biofuel and value-added products manufacture instead of being decomposed into the atmosphere as CO_2_. Combined with solar energy, the chemical energy of wastewater organic matter powers the CO_2_ captured by algae to compensate for the energy consumption of carbon fixation (605 kJ/mol) [222]. Besides, the algal biofilm shows great potential in tolerating toxicants, such as heavy metals [223] and polycyclic aromatic hydrocarbons [224]. This indicates that wastewater from various sources could be used for driving CO_2_ capture by the algal biofilm. Algal biofilm can also be built as a secondary or tertiary treatment unit and inserted as an upgrading module on existing biological treatment units [225,226], making it a flexible technology for wastewater treatment plant construction and upgrading in the future.

Coupling the CO_2_ capture and wastewater treatment by algal-bacteria symbiosis is still premature at present, for most of the studies remain at the laboratory scale. It is necessary to improve the robustness of the technology after scale-up, promote algal-bacteria synergism for optimized CO_2_ capture and conversion efficiency, and further reduce the cultivation footprint [227].

##### 4.2.4.3 CO_2_ conversion with other air pollution gas

Recently, there has also been a great deal of research into the co-conversion of carbon dioxide and other polluting gases, such as H_2_S [142,[228], [229], [230]], volatile organic carbons (VOCs) [231,232], and CH_4_ [143,233]. The efficiency of CO_2_ conversion can be further improved with the aid of other air pollution gases compared to single conversion, and the pollutants can also be converted into other valuable chemicals, so the strategy has both economic and environmental benefits [228,229]. Recently, our group reported a strategy to enhance the photoreduction efficiency of CO_2_ by introducing H_2_S (Fig. 6B) [142]. A well-defined CdS/TiO_2_:Cu hollow sphere with highly dispersed heterointerfaces was designed as a photocatalyst. The hollow structure effectively improved the utilization efficiency of light. More importantly, the existence of H_2_S promoted the consumption of photogenerated holes and the production of photogenerated electrons, thus promoting the production efficiency of CO_2_ into syngas. Besides, H_2_S can also remediate photocorroded cadmium sulfide and extend the service life of heterojunction. H_2_S can also repair photocorroded CdS and extend the service life of heterojunction. At the same time, sulfur and hydrogen elements can also be recovered from the environmental toxicant.

## 5 Challenges and perspectives

**Fig. 7:**
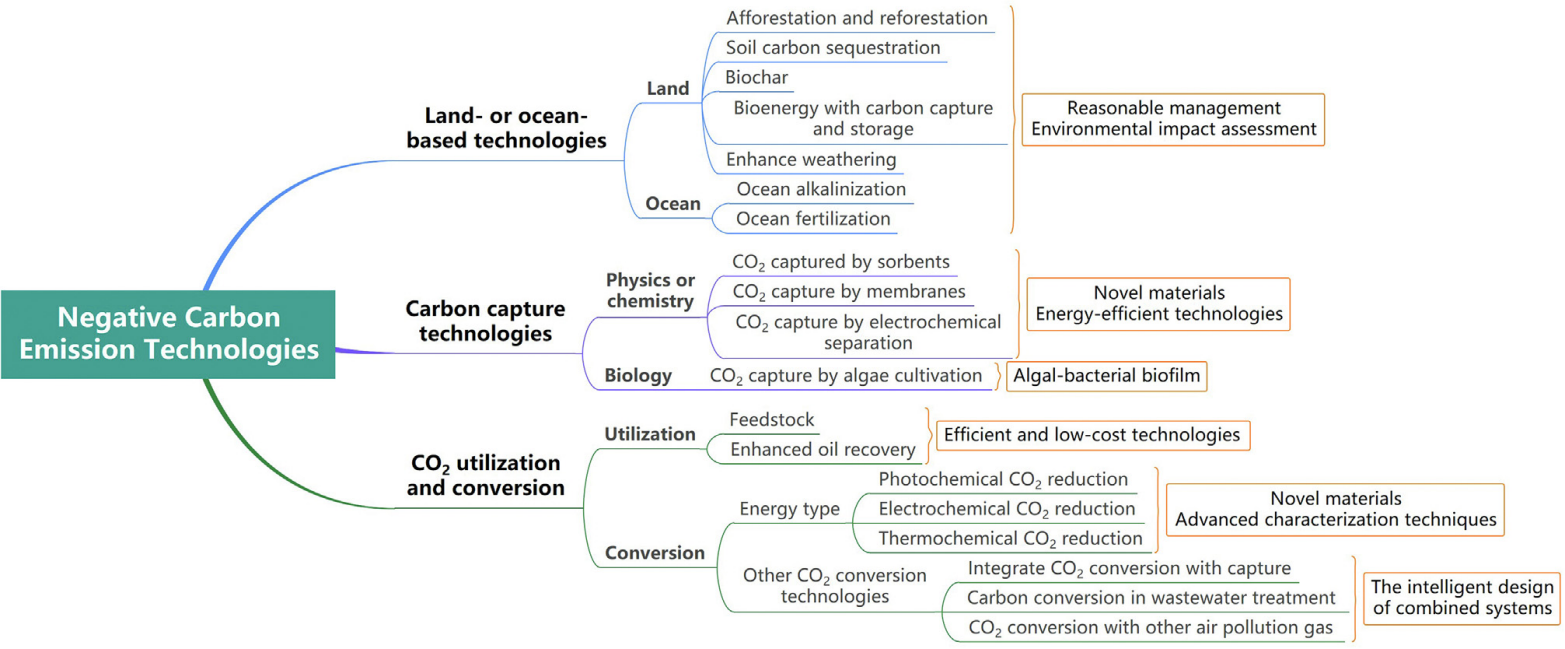
Negative carbon emission technology roadmap to achieve carbon neutrality.

This review summarizes the progress of negative carbon emission technologies over the past few years, including land-or ocean-based technologies, carbon capture technologies, and carbon utilization and conversion. The advantages and disadvantages of these technologies have been summarized and discussed. In brief, the carbon sink processes based on land and ocean can be artificially enhanced by taking physical, chemical, or biological measures. However, the capacity of land and sea is limited and cannot be depended upon indefinitely. Otherwise, it could cause severe ecological damage. Thus, controlling the concentration of CO_2_ in the atmosphere can not only rely solely on the above natural process-based strategies but must combine with the artificial strategies of capture, storage, utilization, and conversion. In addition, the cost of removing CO_2_ from the atmosphere varies widely from a few dollars to hundreds of dollars per ton of CO_2_ for each type of negative carbon emission technology (e.g., USD 45–100/t CO_2_ for soil carbon sequestration and USD 100–300/t CO_2_ for carbon capture), depending on the facilities, energy, raw materials and CO_2_ concentration [14,19,78]. Although CO_2_ utilization and conversion technologies can offset part of the cost, it often requires a high concentration of CO_2_ before utilization and conversion. In the practical application process, researchers should take advantage of these technologies as much as possible, adopt a combination of multiple technology strategies, and consider all the potential risks posed to the ecosystem by negative carbon emissions technologies to achieve carbon neutrality and sustainable development (Fig. 7).

### 5.1 For land- or ocean-based technologies

For land-based technologies, local research and evaluation should be conducted and reasonably planned before implementation. Limited by the land, the improvement of carbon sink for afforestation and reforestation must rely on perfect land management. Conventional plantation management is often characterized by the development of monoculture, which leads to multiple ecological problems, including the loss of biodiversity, nutrients, and soil carbon that have markedly hindered the sustainable development of plantation ecosystems [234]. Conversely, conversion to mixed plantations is proven to improve the utilization efficiency of light, heat, and various other resources, increase soil fertility and provide more ecosystem functions than monocultures [235,236]. It is necessary to develop a rationally mixed plantation to promote cooperation among species, reduce competition within or between species, and achieve optimal ecological benefits. Especially in nutrient-tight places, the effect of reasonably mixed planting could be more obvious, and could also help in understanding the interaction between carbon dioxide for enhancing the carbon sink in the soil. At the same time, the root exudates of plants could also affect soil carbon sequestration, which is mainly due to the close relationship with microbial activities [237]. Soil carbon pool is the largest reservoir in the terrestrial ecosystem, so improving soil carbon sink capacity is of great significance to achieve carbon neutrality. Various long-term sequestration mechanisms should be studied to stabilize soil organic carbon, including fertilization, crop rotation, diversification of planting structure, and other measures that could improve land quality and crop yield.

In addition, as a natural green renewable carbon source, biochar is mainly generated through the thermochemical treatment of various waste sources, including agricultural wastes, food wastes, and manure. A large amount of biochar is produced during pyrolysis or gasification reactions, accompanied by high-value biofuels and syngas production [238]. In this case, from the economic and environmental perspective, the realization of high-value utilization of biomass into biochar is a better choice than the direct conversion of biomass into high-quality porous carbon [57]. The current capacity of biochar to adsorb CO_2_ needs to be further improved to meet the demand for negative carbon emissions. On the one hand, with biomass raw materials restricted by the land, it is necessary to explore the performance and techno-economic analysis of the biological carbon prepared from different raw materials and preparation conditions to find an optimal way. On the other hand, biochar should be modified, including improving the specific surface area and micropore content, the modification of basic functional groups, doping of alkaline earth and alkaline metal atoms, and increasing the hydrophobicity, which is beneficial to enhancing its CO_2_ adsorption properties [239]. It is equally necessary to develop bioenergy technology using nonfood crops, and to plant plants on land unsuitable for crop growth to produce biomass. Bioenergy includes bioalcohol, biocoal, biohydrogen, biogas, and biodiesel [240]. Some of the feedstock used to produce bioenergy is grain, for example, biofuel ethanol that usually comes from the fermentation of the sugars in corn grain [241]. In this case, the competition between bioenergy and food in farmland will intensify with the world population’s growth. There is a need to develop bioenergy technologies that feed from non-food crops. Plants on land unsuitable for crops should also be used to produce biomass [60,242]. More importantly, more efficient methods of producing bioenergy should be developed. Enhancing weathering is also an effective way to reduce atmospheric CO_2_. In the process of adopting this method, the selection of rock powder should take into account its impact on soil and plants to not only achieve the absorption of atmospheric CO_2_, but also improve soil quality and provide nutrients for plants.

Compared with scarce and precious land, the ocean accounts for 71% of the earth’s surface and passively absorbs about 10 Gt of CO_2_ every year [75]. In view of the urgency of current climate change, enhancing the ocean carbon sink cannot be ignored. A few ocean scientists highlight ocean fertilization because adding relatively small amounts of iron in certain seas could increase considerable carbon sequestrations of the ocean at a low financial cost [243,244]. But many scientists also worry that this strategy would lead to unforeseen consequences, just like the catastrophic introduction of rabbits to Australia’s ecology. The safety efficacy and economics of large-scale ocean fertilization have been questioned and debated all the time. Therefore, care should be taken when using any ocean fertilization program for carbon offsets. It is necessary to develop appropriate models to predict the direct or indirect impact of ocean fertilization on the ecosystem, monitor the biological and biogeochemical impact on the ecosystem in real time, and prevent the occurrence of catastrophic hazards [79]. Although ocean alkalization has great potential and application prospects, it does not come without risks. This method might damage the acid-base balance of marine organisms, and introduce toxic heavy metals, seriously affecting the ocean biogeochemical cycle and marine ecosystem services.

### 5.2 For carbon capture technologies

Currently, the applications of carbon capture technology can capture most of the carbon dioxide and reduce the environmental impact since the energy system is still dependent on fossil fuels. However, the existing CO_2_ capture technology could result in significant energy losses in both the capture and separation processes of CO_2_, such as the high energy requirements for the solvent regeneration steps of amine washing at high temperatures. Thus, challenges remain in developing advanced energy-efficient technologies for CO_2_ capture. Carbon capture technology and the photothermal effects of materials could be combined to use solar energy to reach absorbent-regeneration temperatures, reducing fossil fuel demand. Furthermore, a great deal of effort is needed to find the capture agent with a uniform structure and excellent performance. In addition to the need to develop technologies for the capture of high concentrations of CO_2_ emissions, it is also necessary to develop technologies for the capture of CO_2_ in low concentrations. Electrochemical CO_2_ capture has attracted more and more attention due to its outstanding advantages of high efficiency, stability, and flexible operation in theory, but it is still in its infancy, including electrolysis, reversible redox reactions, bipolar membrane electrodialysis, etc. It is also necessary to further optimize the electrochemical capture system to grant it desirable Faraday-driven operational efficiency at low current density due to high electric energy consumption and poor capture efficiency.

Algal-bacterial biofilm is a promising CO_2_ capture technology, with numerous potential strategies to further improve its efficiency in CO_2_ sequestration. First, algal strains that have a short generation time, high tolerance to CO_2_, and efficiency in CO_2_ capture have to be screened and cultivated. Genetic engineering can also be used to design ideal traits in algae. For instance, *Chlamydomonas reinhardtii* that over-expressed H^+^-ATPases successfully survived in a high-CO_2_ milieu (20%), with the photoautotrophic production increased by 3.2 folds [245]. Second, one should develop methods for manipulating the algal-bacterial synergistic effects on CO_2_ capture. Thereinto, adding quorum-sensing molecules, such as N-acyl-homoserine lactone, to regulate the symbiosis is among the most attractive routes [246,247]. Third, optimizing the reactor’s design is pivotal to maximizing solar energy utilization. Although the attached growth mode has reduced the footprint, the inevitable interleaving of attaching surfaces limits the exploitation of the vertical space. Adopting energy-free and robust biohybrid mechanoluminescence [248] or light guide [249] to assist the internal illumination are potential orientations for the 2nd generation of algal biofilm reactors with compact configurations.

### 5.3 For CO_2_ utilization and conversion

How to dispose the captured CO_2_ is a key issue. A simply stored CO_2_ may cost more and have a risk of leakage. CO_2_ utilization is considered a better strategy, which can not only solve the problem of CO_2_ storage but also cover the cost of processes such as CO_2_ capture and transportation. In addition to being used as a feedstock in areas such as food, medicine, electronics, and industrial manufacturing, CO_2_-enhanced oil recovery provides a potential solution to mitigate CO_2_ emissions and climate change. Such techniques may be suitable for high-viscosity oil and low-permeability reservoirs, as well as water-bearing depleted fields [250]. The success of the technology depends on various factors, such as the geology, temperature, pressure of the reservoir, and the physical and chemical properties of the oil. The corrosion of CO_2_ on oil production pipes should also be considered. Therefore, it is necessary to improve the efficiency of CO_2_-enhanced oil recovery, and pilot experiments and feasibility assessments should be carried out to avoid adverse environmental impacts when using the technology.

Inspired by natural photosynthesis, developing efficient CO_2_ conversion technologies for synthesizing value-added chemicals and fuels has become a research hotspot in recent years, and has been considered as a promising strategy for stabilizing the global climate. The most critical challenge is overcoming the inert nature of CO_2_ molecules to achieve efficient CO_2_ reduction at scale, which requires the development of high-performance catalysts with fast kinetics, high selectivity, enduring stability, and low manufacturing cost. The photocatalysis of CO_2_ can be driven by abundant and available solar energy. CO_2_ and water are converted into chemicals and fuels with the assistance of photocatalysts. However, the current efficiency of photocatalytic conversion of solar energy into fuel remains low. Besides, the energy input stability of solar energy is insufficient for the long-term photocatalysis of CO_2_ due to the influence of the climate environment, limiting the industrial use of photocatalytic CO_2_ conversion. Compared with photocatalysis, the electrocatalysis of CO_2_ has been considered an alternative solution since the electricity generated from clean energy can be more stable, and the conversion efficiency may be higher. However, long-term development is needed to reach the industrial application level. Furthermore, MES technology is still in the preliminary stages, and many technical and economic challenges need to be addressed before viable technologies become possible, such as high energy requirements, low productivity, and incompatibility between biocatalysis and electrochemical systems. In addition, although the energy of thermal catalysis mainly comes from fossil fuels, researchers are also vigorously studying the use of solar energy as a source of heat. It is still necessary to improve the efficiency of catalytic reduction to increase the conversion rate of CO_2_. The low selectivity of multi-carbon products is another challenge faced by CO_2_ reduction. In addition to the development of better catalysts for CO_2_ conversion, many studies have focused on CO reduction since most products of CO_2_ reduction are toxic CO [251,252]. Moreover, CO is generally regarded as a key intermediate in the carbon-carbon coupling, so the CO reduction strategy is also promising for producing high-value-added multi-carbon products.

#### 5.3.1 Novel materials

To further improve the performance of CO_2_ reduction conversion, it is necessary to find new material compositions and structures. By means of theoretical calculation, the energy barriers of the reaction intermediates on certain crystal surface of the catalysts are calculated and compared to predict their performance and guide their design. Furthermore, a greater understanding of the reaction mechanisms and pathways is needed to help guide the design of catalysts, particularly the rate-determining step in CO_2_ reduction and surface binding of the reaction intermediates, which can be modulated to accelerate the overall reaction rate. In addition to improving efficiency, the selectivity of products must also be taken into account. CO_2_ reduction can produce not only C_1_ products but also many other products with two or more carbon, which have also gained increasing attention due to their higher added value.

#### 5.3.2 Advanced characterization techniques

In addition, advanced characterization techniques are required for exploring the relationship between the structure and activity of the catalysts, such as X-ray absorption spectroscopy, aberration-corrected transmission electron microscopy, time-resolved fluorescence spectroscopy, and electron spin resonance, which can provide detailed information about the structure of the catalyst and insights about the catalytic process. We will also see increasing *in situ* characterization techniques that provide new clues to the binding configuration and environment of reaction intermediates, such as the *in situ* attenuated total internal reflectance Fourier transform infrared spectroscopy (ATR-FTIR) for identifying reaction intermediates, *in situ* X-ray photoelectron spectroscopy (XPS) for providing the structure and chemical states of the material, *in situ* Raman for determining the adsorption sites of the reaction intermediates, and differential electrochemical mass spectroscopy (DEMS) for progressive or quantitative analysis of the electrochemical reaction gaseous or volatile intermediates and final products.

#### 5.3.3 The intelligent design of combined systems

Integrating CO_2_ capture and electrochemical conversion remains a challenge because of the high energy requirements for capturing CO_2_, especially from the air. The coupling of renewable energy-based capture solutions and CO_2_ value can become a sustainable approach in the future to curb CO_2_ emissions and the use of fossil fuels. In addition, many improvements have been made in the direct reduction of CO_2_ from capture media, especially focusing on improving performance and further studying and optimizing capture and conversion processes. More efficient capture agents and catalysts should be explored to make the capture and conversion process more coupled, improve energy utilization efficiency, and reduce costs.

Wastewater is an inevitable product of human life and industrial production. To achieve carbon neutrality, combining carbon conversion and wastewater treatment is a highly potential approach, and the operation can be done within the existing wastewater infrastructure without requiring additional land. Energy-intensive wastewater treatment plants are transformed into integrated water resource recovery facilities to achieve economic, environmental, and social benefits [141]. The combination of microalgae cultivation and wastewater treatment is a method that can purify water and generate products with added value. Nevertheless, several challenges remain to be addressed. Factors such as light, pH, temperature, and dissolved oxygen in sewage treatment plants may be quite different from laboratory conditions, so pilot studies are needed to explore the impact of a complex environment on the performance of microalgae. It is of significance to screen novel microalgae strains with high biomass productivity that adapt to a wide variety of highly contaminated wastewater and climatic conditions. For example, mixotrophic and heterotrophic microalgae are promising options for treating domestic wastewater, which have significant advantages over phototrophic species. They can grow well and have higher biomass yields in high-load organic carbon wastewater [253]. Besides, advanced genetic engineering and omics methodologies are applied to improve the pollutant removal rate, biomass, and biofuel production of microalgae in wastewater [254]. There also exists a need to develop cost-effective planting and harvesting technologies to produce and recover the biomass of living microalgae without affecting the performance of the algae [255].

In the process of factory production, exhaust gas contains not only CO_2_ but also other air pollution gases, such as H_2_S, which cause serious harm to human health and the environment. Special treatment facilities have been built to treat the polluting gases harmlessly, but are mostly energy intensive and require large supplies of fossil fuels. Therefore, it is necessary to develop a synergistic conversion of CO_2_ and other polluting gases. The transformation mechanism of CO_2_ and other air pollutants should be clarified, and then the reaction devices and reaction pathways should be reasonably designed to achieve the optimal effect of synergistic conversion. In addition, novel catalyst materials should be designed and prepared reasonably to evelate the conversion efficiency to the industrial level. Sustainable development should be achieved by combining renewable energy and recycling valuable chemicals from environmental pollutants.

Although many negative carbon emission technologies have been developed, most of them are carried out in the laboratory, lack the validation data for practical application in the factory, and are not ready for large-scale application. The results of the laboratory scale may not be reproducible in practical applications. For example, the CO_2_ capture efficiency of calcium carbonate absorbent in the laboratory can reach 97%, while the efficiency in the factory was only 21.5% [256,257]. Therefore, the technologies must be verified in pilot plants before large-scale application. Negative carbon emission technologies are of both fundamental and practical significance. Despite some current challenges and difficulties, there is no reason to doubt its huge role and potential. More efforts are required in performance, stability, economy, and sustainability for the future applications of negative carbon emission technologies in achieving carbon neutrality.

## Declaration of competing interests

The authors have declared no conflicts of interest.
